# Mathematical modeling of drug-induced receptor internalization in the HER2-positive SKBR3 breast cancer cell-line

**DOI:** 10.1038/s41598-019-49019-x

**Published:** 2019-09-03

**Authors:** Mirjam Fehling-Kaschek, Diana B. Peckys, Daniel Kaschek, Jens Timmer, Niels de Jonge

**Affiliations:** 1 Institute of Physics, Freiburg University, 79104 Freiburg, Germany; 2 Department of Biophysics, Saarland University, 66421 Homburg, Germany; 3 BIOSS Centre for Biological Signalling Studies, Freiburg University, 79104 Freiburg, Germany; 4 Freiburg Center for Systems Biology (ZBSA), Freiburg University, 79104 Freiburg, Germany; 5 INM – Leibniz Institute for New Materials, 66123 Saarbrücken, Germany; 6 Department of Physics, Saarland University, 66123 Saarbrücken, Germany

**Keywords:** Wide-field fluorescence microscopy, Breast cancer, Mechanisms of disease, Computer modelling, Dynamical systems

## Abstract

About 20% of breast cancer tumors over-express the HER2 receptor. Trastuzumab, an approved drug to treat this type of breast cancer, is a monoclonal antibody directly binding at the HER2 receptor and ultimately inhibiting cancer cell growth. The goal of our study was to understand the early impact of trastuzumab on HER2 internalization and recycling in the HER2-overexpressing breast cancer cell line SKBR3. To this end, fluorescence microscopy, monitoring the amount of HER2 expression in the plasma membrane, was combined with mathematical modeling to derive the flux of HER2 receptors from and to the membrane. We constructed a dynamic multi-compartment model based on ordinary differential equations. To account for cancer cell heterogeneity, a first, dynamic model was expanded to a second model including two distinct cell phenotypes, with implications for different conformational states of HER2, i.e. monomeric or homodimeric. Our mathematical model shows that the hypothesis of fast constitutive HER2 recycling back to the plasma membrane does not match the experimental data. It conclusively describes the experimental observation that trastuzumab induces sustained receptor internalization in cells with membrane ruffles. It is also concluded that for rare, non-ruffled (flat) cells, HER2 internalization occurs three orders of magnitude slower than for the bulk, ruffled cell population.

## Introduction

Many cancer types, including head and neck, stomach, colorectal, pancreatic, glioblastoma, lung, and breast carcinomas, are associated with the mutation or over-expression of the members of the human epidermal growth factor receptor family (HER1-4)^1^. Especially the HER2 over-expressed form of breast cancer, affecting 20% of the patients, has substantially attracted interest in clinical oncology and in the research community. This relates probably to the fact that it was one of the first malignant diseases for which a targeted therapy, namely the humanized, monoclonal anti-HER2 antibody trastuzumab, became available 20 years ago^2^. Compared to standard anti-cancer drug treatments, i.e. cytotoxic drugs without cancer specific molecular targets, trastuzumab combines impressive clinical efficacy with minimized side effects, similar to most other antibody-based targeted drugs^3^. However, the challenging issue of primary and acquired drug-resistance limits the benefits of trastuzumab, and of other biologics^4^. Trastuzumab has several modes of action, including immune mechanisms, such as induction of antibody dependent cellular cytotoxicity (ADCC), and inhibition of HER2 mediated mitogenic signaling^5^. How the drug achieves the latter effect still remains controversial. Proposed are mainly the inhibition of receptor dimerization, and HER2 down-regulation by accelerated endocytosis and degradation^6^. To overcome trastuzumab resistance, more knowledge is needed about its mechanism of action, which, even after 20 years of prescription, is still too fragmentary. In this context it is also desired to take into account the role of cancer cell heterogeneity, which is considered as one of the hallmarks of cancer^7^. By combining single-cell data obtained with an improved HER2 labeling, using a small binding probe, i.e. an anti-HER2 Affibody, and a fluorescent, non-quenchable quantum dot label, and a description by mathematical modeling, we here set out to gain new insights. We focus on the very first effects trastuzumab exerts on HER2 overexpressing breast cancer cells, while accounting for inter- and intra-cellular heterogeneities.

## Results

### Fluorescence microscopy of trastuzumab-induced HER2 uptake

**Figure 1:**
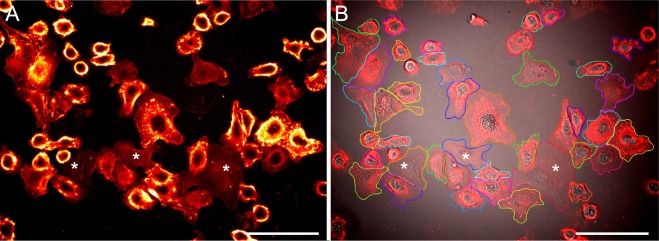
Analysis of membrane-bound HER2 level on SKBR3 cells. (**A**) Membrane-bound HER2 on SKBR3 cells were labeled with an Affibody-quantum dot (QD) to visualize the locations of HER2. Light microscopic image from the QD fluorescence channel acquired for a control group of SKBR3 cells without drug incubation. The typical HER2 distribution, as found in the bulk cancer cells, is characterized by higher HER2 densities (appearing in orange and yellow) on membrane ruffles and at the cell periphery than in flat regions. Three rare flat/resting cells lacking membrane ruffles are marked with asterisks. The QD fluorescence signal is shown in false color (red hot LUT) to better discern the HER2 distribution patterns. (**B**) Overlay of the original red fluorescence channel image, with the corresponding direct interference contrast (DIC) channel image, yielding additional information of the membrane topography. These overlay images were used to manually mark all individual cells, except incompletely imaged cells at the image borders and small spherical cells. The marking resulted in distinctly coloured regions of interest each defining the area used for the measurement of total membrane-bound HER2 per cell, as calculated from the intensity in the QD-channel image. Scale bars: 100 *μ*m.

HER2 is abundant in the plasma membrane of SKBR3 cells, which is a well-established HER2 over-expressing breast cancer cell line and used in numerous *in vitro* studies^8^. To visualize membrane-bound HER2, we applied our previously established two-step HER2 labeling protocol^9,10^. Live SKBR3 cells were first incubated for 10 min with a biotinylated anti-HER2 Affibody. Affibodies are genetically engineered, small bacterial proteins, designed to bind with high affinity to a specific target protein. Functionally they imitate monoclonal antibodies, but they are 10 to 20-times smaller than antibodies. After a subsequent drug incubation, which was omitted for control cells, the cells were fixed and incubated with streptavidin quantum dots (QDs). The protocol ensures a 1:1 labeling stoichiometry between HER2 and QD. The fixation step was necessary to exclude artificial clustering and endocytosis of HER2, inducible by multivalent QD labels^11^. Figure 1A shows the typical QD-fluorescence signature of SKBR3 cells. HER2 is distributed over the plasma membrane, whereby it locally accumulates in membrane ruffles and at the cell edges, consistent with previous studies^9,12,13^. Membrane ruffles are highly motile plasma membrane protrusions at the cell surface. From a top-view on SKBR3 cells, they usually appear elongated, almost worm-like, with a lateral thickness of ~0.5 *μ*m in their short dimension and up to several microns in their long dimension. They are mainly localized towards the periphery of adherently grown cells. In addition, smaller, finger-like protrusions at the cell edges, or a cell surface speckled with dynamic “pimpled” structures, can be observed. Membrane ruffles contain densely polymerized actin filaments, as well as a multitude of actin-binding and -organizing proteins, which are often connected with transducing membrane receptor molecules such as the members of the HER family^14,15^. It should be noted that QDs do not penetrate through the plasma membrane, even after chemical fixation of the cells, the fluorescence signal is thus a measure of the HER2 expression at the plasma membrane only.

**Figure 2:**
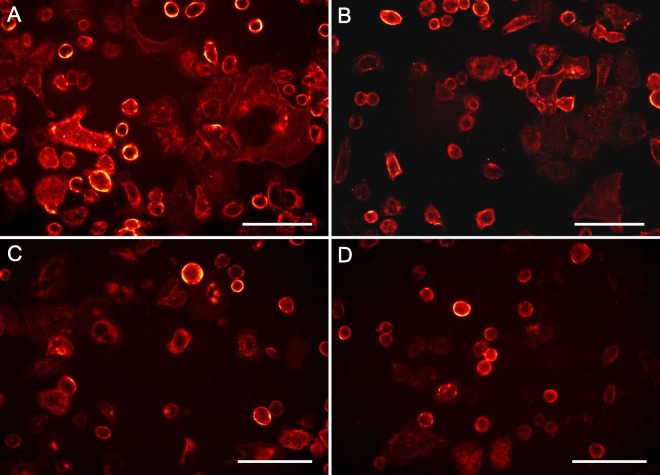
Trastuzumab incubation reduces membrane-bound HER2 levels, and changes the cell membrane topography by abolishing membrane ruffles. Shown are images of the QD-fluorescence channel measuring membrane-bound HER2 represented in false color. (**A**) SKBR3 cells were first incubated for 10 min at 37 °C to label all membrane-bound HER2 with the Affibody, followed by 5 min of incubation with 10 *μ*g/mL of trastuzumab at 37 °C, fixation, and QD labeling. Compared to control cells (see Fig. 1A), the 5 min drug incubation led to a loss of the typically higher HER2 densities on the cell peripheries and on membrane ruffles, as reflected by the absence of membrane areas with orange and yellow color. (**B**) Results of a pulse-chase experiment in which the Affibody-labeled cells were first incubated with trastuzumab as in A, but for 20 min, followed by wash steps and a 2 h chase period in medium at 37 °C, prior to fixation and QD labeling. Compared to A, the level of membrane-bound HER2 has reduced, membrane ruffles have completely disappeared, and a fraction of the cells have reacted by marked constriction. (**C**) Extension of the drug incubation to 1 h (without a subsequent chase time) leads to further reduction of membrane HER2 levels. (**D**) The depicted SKBR3 cells were first incubated for 60 min with trastuzumab, and then labeled with Affibody. This led to similar changes as shown in (**C**), thus, no reversal of the drug effect by reappearing/recycled HER2 was found. The images were recorded and represented with the same settings. Scale bars: 100 *μ*m.

**Figure 3:**
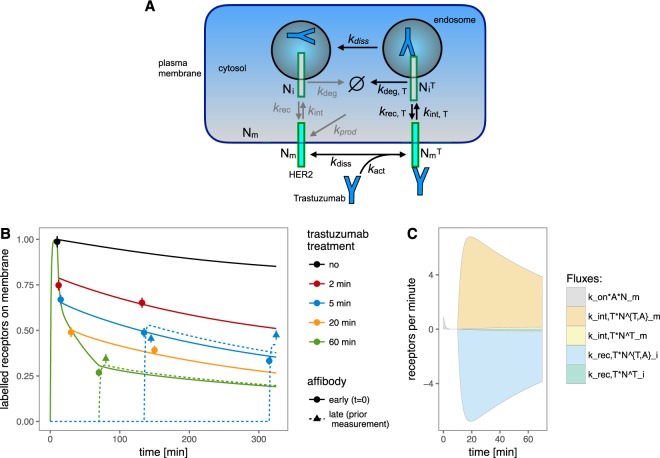
Testing of HER2 recycling model. (**A**) Schematic representation of the recycling model. The Affibody binding process is not shown. The processes represented in grey color were found negligible and removed for the final fitting results. (**B**) Experimental data points and trajectories of the final fit of the recycling model, normalized by dividing by the scaling constant *s*_*c*_. (**C**) Fluxes with relevant contributions in the final fit of the recycling model for the condition with 60 min trastuzumab treatment, normalized by *s*_*c*_. The sign of the flux was chosen such that the backward processes (disassociation and recycling) are negative while all other processes give positive fluxes.

To determine drug-induced membrane clearance of HER2, the cells were exposed to trastuzumab at a concentration of 10 *μ*g/mL. This dose had been shown to exhibit the maximal antiproliferative activity in SKBR3 cells^16,17^, and was also used in other studies of trastuzumab induced HER2 internalization^13,18^ (see Fig. S1). In a first set of experiments, the cells were exposed to trastuzumab for different periods of time, ranging from 2 to 60 min, followed by immediate fixation (e.g., Fig. 2). To examine a possible recycling route of uptaken HER2 back to the membrane, we applied a two-phase technique, called a pulse-chase experiment (Table 1). During the pulse phase of the experiment, the cells were exposed to trastuzumab for different time periods. In the subsequent chase phase, the cells were incubated in drug-free cell culture medium until fixation. Thereafter, single-cell measurements of the mean QD fluorescence intensity allowed the quantification of the remaining plasma membrane-bound HER2. The Affibody was attached to HER2 prior to drug incubation in most experiments. The binding of the Affibody does not hamper the binding of trastuzumab^19^. To make sure that our Affibody incubation did not influence the examined drug-induced internalization process of HER2, we chose a high Affibody concentration, resulting in an Affibody:HER2 ratio of ~50:1, supposed to saturate all HER2 binding sites. Furthermore, we performed four different control experiments. First, the cells were incubated for 60 min just with the Affibody. After this time period, the measured amount of membrane-bound HER2 remained unchanged compared to a control group without any treatment (see Fig. S2). This confirmed that our Affibody incubation did not lead to uptake of HER2. In addition, three other control experiments were performed to test any Affibody influence on the drug effect. In these tests, the cells were first incubated with trastuzumab for different periods of time, and the Affibody incubation was done afterwards, directly before fixation. The results of these controls are represented by triangles in Fig. 3B, they indicate a similar reduction in the membrane-bound HER2 content, compared to the main experiments, in which the Affibody incubation was done before the drug incubation, shown by the round data points in Fig. 3B. Note, that in the main data sets, a slow dissociation of the Affibody from HER2 during the time period of drug incubation, resulted in lower values of membrane-bound HER2. We can thus conclude that under our experimental conditions, the Affibody did not alter the cellular reaction towards the drug. We did not examine the cells for altered gene expression or proliferation patterns, possibly caused by Affibody exposure, because these effects usually need longer time periods to develop than our experimental time frame. Also, possible effects of HER2 signaling changes were not considered, as these were not assumed to change HER2 internalization or recycling behavior after drug exposure.

**Table 1:** Conditions of the main dataset in which the cells are considered as one cell population.

Condition	Affibody	Drug [min]	Chase Time [min]	Nr of Cells
control	0	—	—	218
2 min	0	2	—	223
2 min chase	0	2	120	185
5 min	0	5	—	302
5 min chase	0	5	120	168
5 min chase aff	bm	5	120	188
5 min chase5	0	5	300	218
5 min chase5 aff	bm	5	300	189
20 min	0	20	—	209
20 min chase	0	20	2	202
60 min	0	60	—	198
60 min aff	bm	60	—	176

The conditions differ in the time point of Affibody treatment: either before the experiment (time = 0 min) or before measurement (bm), the drug treatment (none or 2–60 min) and an optional chase time. The number of measured cells per condition is given in the last column.

Cells were first incubated for 10 min with Affibody, then exposed to trastuzumab for 5 min (Fig. 2A), or for 20 min followed by a 2 hour chase period (Fig. 2B), or for 60 min (Fig. 2C). Compared to untreated cells (see Fig. 1A), trastuzumab led to a significant decrease in membrane-bound HER2. To examine a possible recycling of endocytozed HER2, which would lead to a replenishing of HER2 in the plasma membrane, experiments were performed in which the Affibody incubation was done *after* the cells were incubated for 60 min with the drug (Fig. 2D). Thereby, all HER2 receptors in the plasma membrane would be labeled irrespective of their source: recycled or *de-novo* synthesized. As can be seen by comparing the HER2 signal intensities in (Fig. 2C,D), no difference was discernible (see also both green markers in Fig. 3B), thus excluding a significant recycling of internalized HER2 back to the plasma membrane during the 60 min chase period. To examine the existence of a possibly slower recycling process, pulse chase experiments were performed in which the drug incubation was followed by a chase period of 2 or 5 hours, during which the cells were in growth medium without drug. Also in these experiments, no indication for a recycling process was found (compare the positions of the corresponding round and triangle markers in Fig. 3B).

### Analysis of trastuzumab-induced HER2 uptake

Fluorescence microscopy data was acquired from several hundreds of cells for each experimental group. An overview of the experimental groups is shown in Table 1. The data was quantified by measuring the mean QD fluorescence signal intensity per cell as measure of the HER2 membrane density. For this purpose, the outline of each cell was manually indicated in each image and the corresponding mean fluorescence intensity for the QD fluorescence channel was determined using the software of the microscope manufacturer (Leica), see Fig. 1. After background correction, these values were used for calibration of a mathematical model as described below.

### Difference between cell phenotypes

To examine the drug effect in more detail, we considered the presence of different cell phenotypes in the heterogeneous cancer cell population. The single-cell data were thus grouped into distinct phenotypic subpopulations. As was found in a previous study, trastuzumab-induced HER2 uptake predominantly takes place in bulk/ruffled cancer cells, while flat/resting cells without membrane ruffles (examples are marked with an asterisks in Fig. 1A,B) do not exhibit significant uptake^20^. A set of experiments with different timings and controls was performed to determine the difference between flat- and ruffled membrane areas in the drug-induced HER2 clearance from the plasma membrane. In these experiments, the cells were inspected for their ruffling status using direct interference contrast (DIC) microscopy images combined with time lapse imaging, and subsequently grouped into two phenotype-specific groups. One group contained the flat/resting cells, defined as having none or only a single ruffle. The other group included all bulk cells that had more than one ruffle (for details see^20^). The data from these experiments were then used to build a refined mathematical model that included two distinct cell populations with different trastuzumab-induced HER2 uptake rates (see below).

### A mathematical model of receptor recycling

As first approach to evaluate the involved receptor processes, a mathematical model was implemented describing the receptor trafficking processes following a similar approach as described in literature^21^. This model did not consider the cellular heterogeneity of SKBR3 cells, consequently only one population is assumed. A schematic representation of the model is shown in Fig. 3A. According to the model, receptors can either be located in the membrane, denoted as *N*_*m*_, or inside the cell, denoted as *N*_*i*_. There are four processes involved in receptor trafficking:

1. Production of new receptors including their insertion into the plasma membrane with rate *k*_prod_.
2. Internalization of receptors from the membrane into intracellular compartments with flux *k*_int_ · *N*_*m*_.
3. Recycling of internalized receptors back to the membrane with flux *k*_rec_ · *N*_*i*_.
4. Degradation of internalized receptors with flux *k*_deg_ · *N*_*i*_. (Note:The degradation rate refers to the removal of receptors from the pool of receptors that has the capability to recycle back to the membrane. The degradation process itself may take much longer and is not part of this model).

It was assumed that the system was in equilibrium before the Affibody label and drug were added. This allows one to express two rate constants as functions of other parameters: *k*_deg_ = *k*_prod_/*N*_*i*_(*t* = 0) and *k*_int_ = (*k*_prod_ + *k*_rec_ · *N*_*i*_(*t* = 0))/*N*_*m*_(*t* = 0), where *N*_*i*_(*t* = 0) and *N*_*m*_(*t* = 0) correspond to the initial value parameters of *N*_*i*_ and *N*_*m*_. Once trastuzumab with amount *T* was added to the liquid surrounding of the cells, binding of trastuzumab to the receptors took place at the extracellular side of the plasma membrane. Drug binding in turn activated a process that led to the internalization of HER2 receptors with bound trastuzumab^20,22–27^. The exact mechanism leading to the internalization upon drug binding is unknown but was assumed to occur via cross-linking of HER2 homodimers by the antibody^20^. In our model, the binding of trastuzumab to the receptor was included in an activation flux *k*_act_ · *N*_*m*_ · *T* that describes the transition of *N*_*m*_ to the state ${N}_{m}^{T}$, which then transitions to the internalization process governed by the internalization rate *k*_int_.

Note that the actual binding rate of trastuzumab to the receptor was possibly higher than the activation rate. Note furthermore that the activation of the HER2 internalization process should not be confused with the activation of the receptor for signaling. The drug can also dissociate leading to the reverse reaction with flux ${k}_{{\rm{diss}}}\cdot {N}_{m}^{T}$. The receptors with bound trastuzumab ${N}_{m}^{T}$ internalize with a different rate constant *k*_*int*,*T*_ compared to the unbound receptors.

Moreover, the rate constants for recycling and degradation of the internalized receptors with bound trastuzumab ${N}_{i}^{T}$ very likely differed from those of receptors without bound trastuzumab, and were therefore allowed to take different values *k*_rec,T_ and *k*_deg,T_. Similar as in the plasma membrane, trastuzumab can dissociate from the internalized receptor ${N}_{i}^{T}$ once inside the cell. Because data on the latter process are not available, we assumed that it returned to the state *N*_*i*_ with the same rate constant *k*_diss_ as when bound to HER2 in the plasma membrane. To describe the availability of trastuzumab during the time course of the experiment, the trastuzumab amount *T* was implemented as the product of the initial amount of trastuzumab *N*_*T*_ and a switch *s*_on_: *T* = *N*_*T*_ · *s*_on_ with

$${s}_{{\rm{on}}}=\{\begin{array}{ll}0 & {\rm{no}}\,{\rm{Tavailable}}\\ 1 & {\rm{T}}\,{\rm{available}}.\end{array}$$

The switch *s*_on_ also allows to represent the washing off of trastuzumab in case of pulse-chase conditions. During drug treatment, the number of trastuzumab molecules exceeded the number of receptors by a factor of roughly 40, since about 2.5 · 10^4^ L of 69 nM (1 · 10^13^) trastuzumab were added to a dish of about 1.4 · 10^5^ cells with about 2 · 10^6^ receptors per cell (~3 · 10^11^ receptors). The absolute number of HER2 present in the dish was calculated by counting the number of cells from the control group of a typical drug exposure experiment, in a representative, 0.25 *mm*^2^ large area of a cell culture dish, multiplied by a factor of 1000, to account for the total area of 2.5 *cm*^2^, and assuming an average number of HER2 per cell to be 2 · 10^6^ (considering a reported range of 1.4 · 10^6^ to 7.2 · 10^6^ HER2/cell^28,29^). In the mathematical model, the ratio of initial amounts of trastuzumab and receptors was therefore fixed by *N*_*T*_(*t* = 0) = 2.5 · 10^4^ · *N*_*m*_(*t* = 0).

The Affibody state *A* was implemented similar to trastuzumab, as a product of a switch and the inital amount of Affibody *A*_0_ = *A*(*t* = 0). About 1.8 · 10^−4^ L of 0.2 *μ*M Affibody were added, giving a ratio of *N*_*A*_(*t* = 0) ~ 50 · *N*_*m*_(*t* = 0). In summary, *A* was implemented as

$${A}_{0}=\{\begin{array}{ll}0 & {\rm{no}}\,{\rm{Affibody}}\,{\rm{available}}\\ 50\cdot {N}_{m}(t=0) & {\rm{Affibody}}\,{\rm{available}}.\end{array}$$

It was assumed that the presence of the Affibody did not affect any of the other rate constants, i.e. of the HER2 trafficking, activation of trastuzumab-induced HER2 internalization, etc. The original equations describing receptor trafficking were duplicated for Affibody-bound receptors. Additional terms were included to reflect that the Affibody can bind to both receptor states in the membrane, *N*_*m*_ and ${N}_{m}^{T}$, with rate constant *k*_on_ yielding ${N}_{m}^{A}$ and ${N}_{m}^{T,A}$ but cannot bind to internalized receptors. Yet, HER2 with bound Affibody ${N}_{m}^{A}$ and ${N}_{m}^{T,A}$ may internalize yielding ${N}_{i}^{A}$ and ${N}_{i}^{T,A}$. The Affibody can dissociate with rate constant *k*_off_ from both receptors in the membrane (${N}_{m}^{A}$, ${N}_{m}^{T,A}$) and internalized receptors (${N}_{i}^{A}$, ${N}_{i}^{T,A}$). It should be noted, that the amounts of receptors with bound Affibody or trastuzumab were set to zero such that only *N*_*m*_ and *N*_*i*_ were available at the beginning of the experiment.

The full set of ordinary differential equations (ODEs) is given in Table 2. The modeled states *N*_{*m*,*i*}_ are linked to the measured mean fluorescence signal *N*_*obs*_ via the observation function,

$${N}_{{\rm{obs}}}={s}_{c}\cdot ({N}_{m}^{A}+{N}_{m}^{T,A}),$$

since the mean fluorescence signal was proportional to the mean density of Affibody-QD labeled receptors in the membrane. Due to the structure of the differential equations and the observation function, it was possible to simultaneously change the scaling parameter *s*_*c*_ and the initial value parameter *N*_*m*_(*t* = 0) in a way that left the predicted fluorescence signal *N*_obs_ unchanged. We used this freedom of scale to fix *N*_*m*_(*t* = 0) to one, meaning that all receptor numbers *N*_*m*_(*t*), ${N}_{m}^{T}(t)$, *N*_*i*_(*t*), ${N}_{i}^{T}(t)$, ${N}_{m}^{A}(t)$, ${N}_{m}^{T,A}(t)$, ${N}_{i}^{A}(t)$ and ${N}_{i}^{T,A}(t)$ were expressed as multiples of the initial number of receptor on the membrane *N*_*m*_(*t* = 0).

**Table 2:** List of differential equations for the recycling model.

$$\frac{d}{dt}$$N_*m*_ = *k*_prod_ − *k*_act_ · N_*m*_ · T + *k*_diss_ · $${{\rm{N}}}_{m}^{T}-{k}_{{\rm{int}}}$$ · N_*m*_ + *k*_rec_ · N_*i*_ − *k*_on_ · A · N_*m*_ + *k*_off_ · N$${m}^{A}$$
$$\frac{d}{dt}\,$$N_*i*_ = *k*_diss_ · N$${i}^{T}$$ + *k*_int_ · N_*m*_ − *k*_rec_ · N_*i*_ − *k*_deg_ · N_*i*_ + *k*_off_ · N$${i}^{A}$$
$$\frac{d}{dt}\,$$N$${m}^{T}$$ = *k*_act_ · N_*m*_ · T − *k*_diss_ · $${{\rm{N}}}_{m}^{T}-{k}_{{\rm{int}},{\rm{T}}}$$ · N$${m}^{T}$$ + *k*_rec,T_ · $${{\rm{N}}}_{i}^{T}-{k}_{{\rm{on}}}$$ · A · N$${m}^{T}$$ + *k*_off_ · N$${m}^{T,A}$$
$$\frac{d}{dt}\,$$N$${i}^{T}$$ = −*k*_diss_ · N$${i}^{T}$$ + *k*_int,T_ · $${{\rm{N}}}_{m}^{T}-{k}_{{\rm{rec}},{\rm{T}}}$$ · $${{\rm{N}}}_{i}^{T}-{k}_{{\rm{\deg }},{\rm{T}}}$$ · N$${i}^{T}$$ + *k*_off_ · N$${i}^{T,A}$$
$$\frac{d}{dt}\,$$N$${m}^{A}$$ = −*k*_act_ · N$${m}^{A}$$ · T + *k*_diss_ · $${{\rm{N}}}_{m}^{T,A}-{k}_{{\rm{int}}}$$ · N$${m}^{A}$$ + *k*_rec_ · N$${i}^{A}$$ + *k*_on_ · A · N_*m*_ − *k*_off_ · N$${m}^{A}$$
$$\frac{d}{dt}\,$$N$${m}^{T,A}$$ = *k*_act_ · N$${m}^{A}$$ · T − *k*_diss_ · $${{\rm{N}}}_{m}^{T,A}-{k}_{{\rm{int}},{\rm{T}}}$$ · N$${m}^{T,A}$$ + *k*_rec,T_ · N$${i}^{T,A}$$ + *k*_on_ · A · $${{\rm{N}}}_{m}^{T}-{k}_{{\rm{off}}}$$ · N$${m}^{T,A}$$
$$\frac{d}{dt}\,$$N$${i}^{A}$$ = *k*_diss_ · N$${i}^{T,A}$$ + *k*_int_ · $${{\rm{N}}}_{m}^{A}-{k}_{{\rm{rec}}}$$ · $${{\rm{N}}}_{i}^{A}-{k}_{{\rm{\deg }}}$$ · $${{\rm{N}}}_{i}^{A}-{k}_{{\rm{off}}}$$ · N$${i}^{A}$$
$$\frac{d}{dt}\,$$N$${i}^{T,A}$$ = −*k*_diss_ · N$${i}^{T,A}$$ + *k*_int,T_ · $${{\rm{N}}}_{m}^{T,A}-{k}_{{\rm{rec}},{\rm{T}}}$$ · $${{\rm{N}}}_{i}^{T,A}-{k}_{{\rm{\deg }},{\rm{T}}}$$ · $${{\rm{N}}}_{i}^{T,A}-{k}_{{\rm{off}}}$$ · N$${i}^{T,A}$$
$$\frac{d}{dt}\,$$T = −*k*_act_ · N$${m}^{A}$$ · T + *k*_diss_ · $${{\rm{N}}}_{m}^{T,A}-{k}_{{\rm{act}}}$$ · N_*m*_ · T + *k*_diss_ · N$${m}^{T}$$
$$\frac{d}{dt}\,$$A = −*k*_on_ · A · N_*m*_ + *k*_off_ · $${{\rm{N}}}_{m}^{A}-{k}_{{\rm{on}}}$$ · A · N$${m}^{T}$$ + *k*_off_ · N$${m}^{T,A}$$

The HER2 receptor *N* is labeled with suffix *m* or *i* indicating its location either on the membrane or internal. Bound Trastuzumab *T* or Affibody *A* are indicated by the superscripts *T* and *A*.

The model parameters were estimated from the data summarized in Table 1 using the maximum likelihood method. Optimization of the log-likelihood function from different initial guesses for all parameters revealed the existence of several local optima that describe the data almost equally well in terms of their log-likelihood value. To diminish the number of different solutions describing the data, additional assumptions were introduced to reduce the model complexity.

As a first step, solutions giving a larger fraction than 5% of internalized receptors (*N*_*i*_/*N*_*m*_) initially were omitted, since most receptors were assumed to reside in the plasma membrane after the initial serum starving applied prior to the experiment; values of about 2.5% internalized HER2 under control conditions have been reported in literature^30^. The model was further simplified by imposing a limit range on the Affibody binding parameter *k*_on_, which excluded several solutions. Boundaries for the Affibody binding rate were extracted from measurements of cellular uptake published elsewhere^31^, leading to the range $0.5\lesssim {k}_{{\rm{on}}}\cdot {A}_{0}\lesssim 4$ 1/min. The dissociation rate *k*_off_ was estimated consistently between the different solutions within a narrow range of 0.5 · 10^−3^ to 1 · 10^−3^ 1/min. This value is consistent with the value of 5 · 10^−3^ 1/min (half-life ${\tau }_{\frac{1}{2}}=2.2\,{\rm{h}}$) in the literature^31^. A common feature of the remaining solutions was that an upper bound for the receptor synthesis process *k*_prod_ < 1.5 · 10^−4^ 1/min was derivable. A half life of ${\tau }_{\frac{1}{2}}=9.4\,{\rm{h}}$ was reported for HER2 receptors on a non-starved HER2 positive N87 gastric cancer cell line^32^. Starvation is expected to reduce this value by about 50% (ref.^33^). Accordingly, the production rate in starved N87 cells would be *k*_prod_ ~ 6 · 10^−4^ 1/min, which is in a similar range as the upper bound we found. On the other hand, synthesis rates between N87 and SKBR3 cells do not necessarily have to coincide. For a production rate *k*_prod_ tending to zero, also the degradation process *k*_deg_ · *N*_*i*_ must decrease because, otherwise, the receptor distribution in equilibrium would change. Omitting both, production and degradation process from the model did not deteriorate the fit. Therefore, in favor of the smaller model, these two processes were neglected.

Finally, three solutions remained with different parameters but no significant difference in the goodness of fit, measured by a likelihood ratio test. The main difference between the solutions was whether or not recycling and internalization of receptors was needed in the absence of trastuzumab.

Again, in favor of the smaller model, both, recycling and internalization of receptors that had not bound trastuzumab were removed from the model. The other solutions are discussed in the Supplementary Information. A schematic representation of the final model is shown in Fig. 3A, whereby all processes neglected in the final results are shaded gray.

The experimental data and the fitted trajectories of the final model are shown in Fig. 3B. The trajectories of the model are in close agreement with the datapoints. In particular, the observed two time-scales are also present in the model, a fast reduction of receptors in the membrane within the first five minutes followed by a slower reduction of receptors from about 5 to 60 minutes are reproduced. The parameter values and their 95% confidence intervals as being computed by the profile likelihood method (shown in Supplementary Information) are reported in Table 3. The time-resolved flux contributions for all processes are shown in Fig. 3C. All parameters are identifiable apart from the drug-induced internalization and recycling rates, where only lower bounds were defined. It turns out that the strong fluxes are needed to describe the two time-scales for the reduction of receptors in the membrane. The values of the drug-induced internalization and recycling rates are extremely large, predicting that on average, the whole receptor population recycles back and forth from the membrane five times per minute. This is a non-physiological fast rate. For example, the fastest known receptor recycling rates occur in cells in the so-called rapid recycling pathway in the endosomal sorting machinery^34–37^, and these are at least two orders of magnitude slower than the value fitted here. Since it is non-physiological that the whole receptor population is renewed several times per minute, we conclude that the model does not accurately describe the involved biological mechanisms. It was therefore questioned, whether other processes should be implemented in the model to alternatively describe the two time-scales. A suggested model extension is presented in the next section.

**Table 3:** Parameter values obtained by the model fit for the first, basic model (Model A) and second, extended model (Model B).

Parameter	Model A	Model B	Unit
*k*_act_ · *T*_0_	0.39 ± 0.09		1/min
*k*_act,R_ · *T*_0_		0.4 ± 0.1	1/min
*k*_act,F_ · *T*_0_		0.41^*^	1/min
*k*_diss_	2.3 ± 0.8	5.0 ± ${\,}_{2.0}^{2.9}$	10^−2^/min
*k*_on_ · *A*_0_	1.0 ± ${\,}_{0.7}^{20}$	1.0^**^	1/min
*k*_off_	6.4 ± ${\,}_{4.1}^{5.3}$	6.8 ± 4.1	10^−4^/min
*k*_int,T_	12.8 ± ${\,}_{12.1}^{\infty }$		1/min
*k*_int,RT_		49 ± ${\,}_{47}^{\infty }$	1/min
*k*_int,FT_		0.0126 ± 0.0028	1/min
*k*_rec,T_	20 ± ${\,}_{19}^{\infty }$	NA^***^	1/min
*k*_deg,T_	0.87 ± ${\,}_{0.79}^{0.90}$	NA^***^	10^−2^/min
*s*_*c*_	529 ± 26	535 ± 27	
N_*m*_(*t* = 0)	1^****^		
N_*F*_(*t* = 0)		0.62 ± 0.065	
N_*R*_(*t* = 0)		0.38^****^	

For the parameters related to the drug or Affibody binding, effective rates (*k*_act_ · *T*_0_, *k*_on_ · *A*_0_) with *T*_0_ = *T*(*t* = 0) and *A*_0_ = *A*(*t* = 0) are given. The uncertainties correspond to 95% confidence intervals obtained from the parameter profiles. No upper bounds were found for the *k*_int,T_, *k*_rec,T_ and *k*_int,RT_ parameters, therefore ∞ is given for the upper uncertainty.

*The difference between *k*_act,R_ and *k*_act,F_ was estimated to zero, therefore *k*_act,R_ = *k*_act,F_.

**In case of Model B, the *k*_on_ parameter was non-identifiable and fixed to the value from Model A.

***The second, extended model B was fitted without recycling and degradation processes.

****The initial value for the number of receptors (Model A: N_*m*_, Model B: N_*R*_ + N _*F*_) on the membrane is fixed to one.

### Extended model with membrane ruffles

Because the first model resulted in a non-physiological high recycling rate of trastuzumab-bound HER2, we developed a second model, extended by consideration of two distinct cell phenotypes, reflecting observations that SKBR3 cells can be grouped into different subpopulations with respect to the phenotype of their membrane topography. For this purpose, the entire SKBR3 population was divided into a large fraction of bulk cells, having a highly dynamic, ruffling plasma membrane, and a small fraction (~6%) of resting, or flat cells, with no or at most one membrane ruffle. As we have shown that homodimeric HER2 is only found in the ruffled bulk cell subpopulation^9^ we concluded that a direct correlation links the existence of ruffles with homodimeric HER2 and the efficiency of the drug to induced HER2 down-regulation from the plasma membrane^20^. The main experimental dataset (Table 1) was re-evaluated discriminating between the flat and ruffled cell phenotypes for the first few time points up to 5 min. It was impossible to make this discrimination for time points larger than 5 min because trastuzumab incubation led to a vanishing of the ruffles in bulk cells. To track the cells over a longer drug treatment period of 60 min, a new experimental series was performed in which the cell phenotype was determined before the drug was added (Table 4), and the individual cells were tracked through the course of the experiment. At the end of the experiment, membrane-bound HER2 was QD-labeled and quantified.

**Table 4:** Conditions of the additional dataset in which the cells were considered either as ruffled, in case more than one membrane ruffle was found, or as flat, in case no or at most one membrane ruffle was found.

Condition	Cell Type	Drug [min]	Nr of Cells
control flat	flat	—	38
control ruffled	ruffled	—	69
min flat	flat	60	66
min ruffled	ruffled	60	74

The cells were incubated with Affibody before the experiment, and then incubuated with drug for 60 min drug. A control experiment without drug was also performed. No chase period was applied. The number of measured cells per condition is listed in the last column.

**Figure 4:**
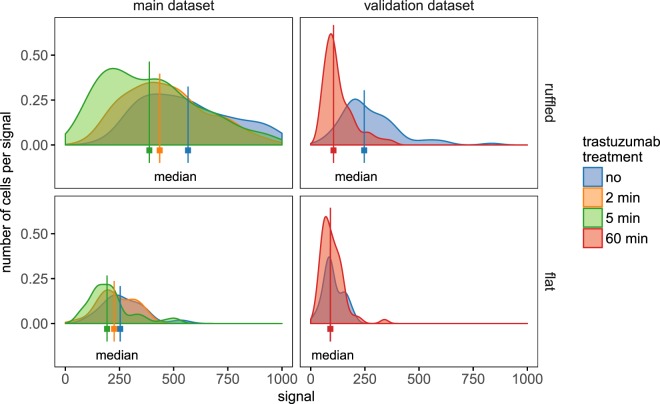
Normalized HER2 bound QD fluorescence intensity distribution. Shown is the difference in trastuzumab induced HER2 removal from the membrane between bulk cells, characterized by more than one membrane ruffle per cell (upper graphs), and flat cells, with no or only one membrane ruffle per cell (lower graphs). The main data set (left side) depicts the early reactions after 2 min (orange), and 5 min (green) of drug incubation, compared to control cells without drug incubation (blue). The validation dataset (right), determined after 60 min of drug incubation, shows a marked shift of the median of the ruffled bulk cell population towards median values of the flat cell population, indicating a more than 50% reduction of membrane-bound HER2 within the bulk subpopulation. The mean HER2 signal distribution of the control flat cells started at already lower mean intensity values, compared to the ruffled cells. At early time points these cells exhibited only small, negligible shifts towards lower intensity values, after 60 min this subpopulation completely failed to show a difference towards the untreated flat cell population.

Figure 4 shows the normalized QD fluorescence intensity indicating membrane-bound HER2 as obtained from the single-cell, background corrected, distribution histograms of the mean QD fluorescence intensities for both datasets. In the main dataset, already at the first examined time points of 2 and 5 min, the flat and the ruffled cells show a different reponse to the drug. This effect is even more pronounced in the additional dataset (see Fig. 4) after the 60 min treatment duration, when the bulk cells have clearly decreased their membrane-bound HER2 levels. In the flat control cell population without drug incubation, a lower mean HER2 fluorescence intensity is observed than for the ruffled control cells. At the early time points, only a slight decrease is observed for the flat cells, after 60 min of drug incubation no difference is found versus the control flat cells. These cells are thus resistant against drug-induced HER2 internalization.

Ruffled cells contain ruffled and flat membrane regions. As the receptors in the flat regions of bulk cells are expected to behave similar to receptors on entirely flat cells, the advanced model distinguishes between receptors from flat and ruffled *regions* rather than between flat and ruffled *cells*. The overall proportion of receptors from flat regions in the whole SKBR3 cell population is thus higher than the 6% share of flat cells. The exact value is estimated together with the other model parameters.

**Figure 5:**
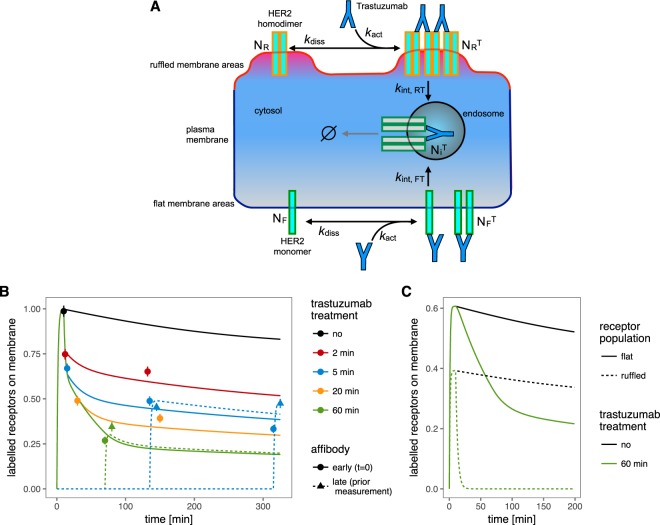
Testing of extended model including SKBR3 cancer cell heterogeneity. (**A**) Schematic representation of the model with ruffled and flat receptor populations on the membrane. (**B**) Fitted model trajectories with data points. (**C**) Model trajectories separated for the contributions of ruffled and flat regions for the control and 60 min trastuzumab treatment conditions.

In the advanced model, HER2 receptors from the ruffled and the flat membrane regions are denoted as *N*_*R*_ and *N*_*F*_, see Fig. 5A. The observed receptors on the membrane thereby consist of contributions of both populations: ${N}_{obs}={s}_{c}\cdot ({N}_{R}^{A}+{N}_{R}^{T,A}+{N}_{F}^{A}+{N}_{F}^{T,A})$, this is the receptors with and without trastuzumab (T) in the ruffled regions (R) plus the receptors with and without trastuzumab in the flat regions (F). Receptors can only contribute to the observation if they have bound the Affibody (A). Same as for the first, basic model, the freedom of scale is used to fix *N*_*R*_(*t* = 0) + *N*_*F*_(*t* = 0) to one. This means that *N*_*F*_(0) and *N*_*R*_(0) already reflect proportions of the total receptor population.

The drug binding to the receptors in flat and ruffled regions is fitted independently, the disassociation however is assumed to be the same. The increase to four different receptor states on the membrane also leads to an increase of different internal states and their corresponding trafficking processes. Although different and independent recycling processes for the different receptor populations can not be excluded, a straightforward implementation of all possible reactions with independent rate parameters would lead to a heavy over-parameterization of the model. Therefore, the model reductions found for the first, basic modeling approach, i.e. omission of receptor production and degradation, were also adopted for the second, extended model. The second model reaches out to find an answer to the possibility of fast recycling of the internalized receptor. As a radical approach, the model was tested under the restriction that no recycling process is allowed. In this case, also the degradation process can be neglected from the model since no interactions are left for the internalized receptors that would influence the receptor population on the membrane. The model was thereby reduced to describe drug-induced receptor internalization on two time-scales.

A schematic representation of the extended model is provided in Fig. 5A. The Affibody binding rate *k*_on_ was fixed to the value found for the previous fit, given in Table 3. The extended model was calibrated based on the main experimental data set and fits this data equally well as the model with fast recycling, indicated by the trajectories in Fig. 5B.

According to the model fit, *N*_*F*_(0) = 62 ± 10% of all membrane-bound HER2 present in the whole SKBR3 cell population are attributed to the flat membrane regions. The estimated internalization rate for receptors in ruffles is about a factor 1000 faster than for those in flat membrane regions. In contrast, the association rates for receptors in both regions are estimated to the same value and need no distinction. The model parameters of the final, reduced model are reported in Table 3.

In earlier studies we found HER2 on flat membrane regions to exist in the monomeric state^9^, and resisting trastuzumab induced internalization^20^. Although two HER2 monomers can be forced into a dimer by a commonly bound, bivalent molecule of trastuzumab^27^, these dimers seem to be too small to effectively induce their endocytosis. In contrast, the 38% fraction of HER2, located in ruffled membrane regions, and consisting for a large part of HER2 homodimers, can be linked by trastuzumab into large, chain-like oligomers. Compared to the very low constitutive internalization rate of HER2 in control cells^30,38^, the supposed trastuzumab-linked chains of HER2 homodimers can quickly start endocytosis, with a 1000-fold increased internalization rate. It is known that cross-linking with antibodies redirects receptors from a recycling into a degradation pathway, a switch that depends on the size of the cross-linked oligomers^39^, with larger oligomers likely being too bulky to be included in the tubules of the endocyctic recycling compartment^40^. Our data of sustained HER2 clearance from ruffled cells, confirmed by the results from experiments with Affibody labeling after long-term drug incubation (see Fig. 5B), are in complete agreement with these findings. The combined effect of the two different internalization rates is divided in Fig. 5C where the model prediction is shown separately for the two receptor populations in case of the 60 min trastuzumab treatment.

To test whether the data could be described even more accurately, we reintegrated trastuzumab-induced recycling and degradation processes and estimated the parameters again. It turned out that the additional parameters could not significantly improve the fit. The question if a fast HER2 recycling process would be necessary to explain the data was thereby answered negative. It can thus be concluded that after trastuzumab-induced HER2 internalization, no fast recycling process is needed to explain the experimental results.

Judging from the size of the error bars in Fig. 5B, it is imaginable to find models that fit the data more accurate. However, it should be kept in mind that the data points do not all correspond to the same sample or batch of SKBR3 cells. The error bar reflects the standard error of the mean fluorescence of each sample but does not cover inter-sample variability. Cancer cells are continually evolving, a characteristic that is responsible for the typical tumor cell heterogeneity and also accounts for differences between cell batches derived from the same cell line or tumor, but at different passage numbers, respect. different times^41,42^.

The model, without recycling process, was validated based on the additional data set (Table 4) which was to this point not used for model calibration. The ratio of the number of membrane receptors after 60 min trastuzumab treatment and before has been quantified for both cell populations. The values with their 95% confidence interval are reported in Table 5.

**Table 5:** Ratio of the number of membrane receptors after 60 min trastuzumab treatment and before for a sub-population of flat cells and ruffled cells for the validation dataset and the model prediction calibrated on the main dataset.

cell population	data ratio $\frac{{{\boldsymbol{N}}}_{{\boldsymbol{m}}}({\boldsymbol{t}}={\bf{60}})}{{{\boldsymbol{N}}}_{{\boldsymbol{m}}}({\boldsymbol{t}}={\bf{0}})}$ + [95% conf. int]	model prediction
flat	0.94 [0.78, 1.13]	0.66
ruffled	0.44 [0.35, 0.54]	0.29

The observed ratios indicate a strong internalization in ruffled cells whereas the number of membrane receptors on flat cells does not significantly change. The observed ratios are compared to the model prediction for cells with 100% flat regions and cells with a mixture of 60% of HER2 in flat and 40% in ruffled regions, as found by parameter estimation in the main dataset. Although the model correctly predicts a stronger internalization in ruffled cells than in flat cells, the predicted ratios are significantly smaller than observed in the validation dataset. A possible cause could be the above mentioned variability between cancer cell populations of different batches of the cell line over time, affecting the phenotype of ruffling behavior or the usuage of the same activation flux for ruffled- and flat cells, although a different flux would possibly be needed but was not implemented to avoid over-paramerization.

## Discussion

An important hypothesis about the mechanism of the HER2 targeting antibody-drug trastuzumab involves drug-induced HER2 down-regulation. Although the general mechanism of antibody-induced clustering and endocytosis of membrane receptors is known since the early 1980’s^43^, and has been shown to also apply for several HER2 antibodies^44,45^, accumulation of conflicting results hampered a clear consensus in the case of trastuzumab. Already the first effect of trastuzumab, after binding its target on the cell membrane, is still a vivid matter of debate. While a down-regulation of HER2 signaling as a consequence of trastuzumab binding is generally well accepted, it remains controversial how this effect is achieved. Several studies reported a lack of internalization of HER2 and/or a fast recycling of HER2 back to the membrane^13,18,23,46–48^. These studies are challenged by publications demonstrating convincing data of substantially and prolonged HER2 localization in intracellular vesicles after incubation with trastuzumab^22,24–26,44,48–50^. Our own data support the latter results^20^. The discrepancy between those groups of studies is possibly explained by methodological limitations.

The first issue concerns fluorophore self-quenching, an effect appearing when fluorophores come into close proximity^51^. In experiments using fluorophore-labeled antibodies, this effect has been shown to bias the quantification of internalized HER2^52^. Our hypothesis is that trastuzumab rapdily cross-links HER2 homodimers into large oligomers at the extracellular side of the plasma membrane, that are subsequently up-taken via endocytosis. HER2-bound fluorophores will thus be present in endosomes in which they will become highly condensed, leading to self-quenching and thus an underestimation of internalized HER2^52^. Self-quenching might be further amplified in case antibodies are used containing a multiple of dye molecules per antibody^53^, and when polyclonal secondary antibodies are used.

The second technical pitfall relates to the cooling of live cells on ice, as is done in many studies, especially those using flow cytometry. Cooling mammalian cells to 4 °C can induce major changes in the cytoskeleton through loss or damage of actin^54^. Prolongued cold incubation of cells is known to be responsible for several unwanted effects, such as artifactual immobilization of membrane receptors, artifactual cross-linking, and non-specific binding of nanoparticle-antibody probes, applied as specific labels for membrane receptors^55^. A significantly changed binding behavior of Pertuzumab, another therapeutic, monoclonal anti-HER2 antibody, was observed on cooled cancer cells^56^. Also in case of the closely related HER1 (EGFR) receptor, cooling the cells on ice induced marked changes in its membrane distribution^57^. Results from our control experiments, examining the cooling of SKBR3 cells on ice for a 60-min period, followed by a rewarming period to 37 °C, during which trastuzumab was applied, revealed changes in the HER2 plasma membrane distribution after the cooling period and, more importantly, a marked attenuation of the drug effect, compared to cells continuously kept at 37 °C before and during drug incubation (see Fig. S3).

A third problematic bias can be introduced through the use of rather bulky antibodies as labels for HER2, because antibody labeling is prone to the risk of steric hindrance^58,59^. In the case of HER2, this can reduce HER2 label density, and thus lead to different HER2 labeling efficiencies between membrane regions of dense HER2 occurrence, such as ruffles and endosomes, compared to flat membrane regions. In addition, when cells are fixed with formaldehyde before fluorophore conjugated trastuzumab is applied to label HER2^53^, the risk of measurement bias is even higher, because trastuzumab’s binding kinetics dramatically deteriorate on chemically fixed HER2^60^.

Our approach circumvented the above-mentioned experimental pitfalls. To alleviate any bias of the quantification of HER2 fluorescence caused by intravesicular fluorophore self-quenching^51^, we measured the remaining amount of membrane-bound HER2^46^. Moreover, we used adherent, sub-confluently grown, living cells, and a continuous experimental temperature of 37 °C, thereby preserving the local environment of the cells (e.g. cell density) and normal physiological properties of adherent cells. Secondly, the label consisted of an anti-HER2 Affibody, which is 10 times smaller than an antibody and not hampering the binding of trastuzumab^19^. Only at the end of the experiment, thus after the drug incubation, and in some cases after an additional chase period, the cells were chemically fixed. Fixation was necessary to prevent artificial HER2 clustering, which can be induced on live cells by multivalent QDs. This two-step labeling of membrane-bound HER2 with QDs allows direct quantification due to its one-to-one labeling ratio with HER2^9^, and in addition, it minimizes the risk of fluorophore self quenching. Single-cell datasets, reflecting the actual membrane levels of HER2, were collected at different time points of drug treatment. To examine a possible recycling mechanism a set of experiments with the drug incubation and different chase periods performed prior to the Affibody labeling was performed (see triangle markers in the graph of Fig. 5B). Another set of experiments was performed to examine possible differences in the drug effect on monomeric or homodimeric HER2. To this aim, the SKBR3 cell population was divided into two subpopulations, depending on the presence of membrane ruffles, which are the only regions were HER2 exists as homodimer.

The descriptive power of mathematical models of biological processes with ordinary differential equations (ODEs) critically depends on the quality of the underlying biological data. While the model structure itself is generic and has been used in the past to study HER2 trafficking e.g. in epithelial cells^21^, the parameters can vary considerably between different cell types and different experimental conditions. Difficulties in comparing data and conclusions gained from different HER2 (over)expressing cell lines can result from large variations between the absolute HER2 expression levels and different ratios between HER2 and its possible heterodimerization partners, mainly HER1 (EGFR) and HER3^61^. According to our hypothesis, all conditions lowering the share of HER2 homodimers in the plasma membrane will probably reduce trastuzumab induced HER2 internalization. Several groups, including us, have shown that HER2 receptors in over-expressing cells accumulate in ruffled membrane regions^12,13,62,63^. Remarkably, these highly dynamic structures are the exclusive regions where we found HER2 in a homodimeric conformation^9^, responding within a few minutes to trastuzumab by disappearance^20^. The degree of membrane ruffling in HER2 positive cells often correlates with the absolute level of HER2 over-expression^12^, but, there are also reports on breast cancer cell lines with similar HER2 levels, but different degrees of membrane ruffling^64^. Trastuzumab is able to bind to both, monomeric and homodimeric HER2^27^, but the fate of the two types of HER2-trastuzumab complexes differ largely. It is highly probable, that in regions with a high density of HER2 homodimers, trastuzumab creates large, chain-like oligomers, consisting of alternating HER2 homodimers and trastuzumab molecules, which are able to initiate their uptake by endocytosis. Trastuzumab binding to HER2 monomers or heterodimers can only lead to smaller multimeric complexes, lacking the ability of initiating the endocytosis mechanism^40^. The situation is even more complicated by the interplay between HER2 and HER1, which is demonstrated by EGF-induced enhancement of dynamic membrane behavior^65^, the absolute level of HER2 surface expression, and HER2 turn-over rate^66^. These effects depend on the concentration of EGF in the surrounding medium or buffer, as well as the actual cell density, providing EGF for paracrine and autocrine stimulation^67^. The gathering of quantitative results on drug induced HER2 uptake, even when performed with the same cell line, is thus highly sensitive to a multitude of experimental conditions. Earlier published models on trastuzumab-induced HER2 internalization^53,61,68,69^, fall short with respect to the above described technical pitfalls of fluorophore self-quenching, artifacts due to prior cell detachment, fixation preceding labeling, and low temperature effects. Not surprisingly, all these models implement substantial HER2 recycling rates, probably because they would not fit without this assumption.

Apart from these technical hurdles, a common weakness in the design of most studies examining the effect of trastuzumab on HER2 internalization is the neglect of cellular heterogeneity, nowadays considered as hallmark of cancer^70^. Heterogeneity within the overall cell population is reflected by the existence of for instance rare resting/dormant cancer cells^71^. In addition, heterogeneity can also be found at the sub-cellular level, for instance in the form of flat and ruffled membrane regions. Through individual single-cell sorting of SKBR3 cells into ruffled bulk cells, or flat cells, we were able to build a second, extended model that considered the different conformations of HER2 as homodimers and monomers. This model showed an improved descriptive quality compared to the first model, build without any differentiation of SKBR3 cells into subpopulations. We did not include HER2 heterodimers with other members of the receptor family, because of the several-fold lower expression levels of these other HER family members in SKBR3 cells. The ratio of expressed HER2 versus expressed EGFR under our experimental conditions, including serum starvation, is around 20^29^, and the ratio of HER2:HER3 is even 40, HER4 expression is below the detection limit^72^. These proportions leave HER2 heterodimers a minor fraction compared to HER2 homodimers^73^.

Focusing exclusively on the quantities of membrane-bound HER2, our data showed a robust and sustained HER2 down-regulation after trastuzumab incubation. Any significant HER2 recycling process would have resulted in the attenuation or reversal of this down-regulation, or the recycling parameter would have to be set in a non-physiologically high range. By considering HER2 heterogeneity, our second, extended mathematical model allowed eliminating a recycling process without deterioration of the data description.

## Conclusions

Our data show that trastuzumab induces a sustained down-regulation of membrane-bound HER2. The extent of this decrease depends on the exposure time, ranging from 30% average reduction after only 2 minutes to about 60% after 60 minutes. Considering the individual cells, the degree of HER2 reduction strongly depends on the existence of HER2 homodimers in membrane ruffles. Furthermore, the data indicate a negligible restocking of HER2 on the membrane.

Our first, basic model is capable of explaining the observed behavior without consideration of cellular heterogeneity. However, this model requires the implementation of extremely high internalization and recycling rates, two orders of magnitude higher than known rates for recycling. Because the fitted exceptionally high recycling rate appears to be non-physiological, we developed a second, extended model that considers the spatially segregated presence of HER2 in different functional states, i.e. homodimeric and monomeric, preferentially located on ruffled, respectively flat membrane regions^9^. Due to their different susceptibility to chain-like cross-linking by trastuzumab, these two functional states of HER2 have highly different internalization rates. Based on the estimated parameters, the extended model explains the observed data for physiologically realistic process rates. It explains why binding of the same drug to HER2 in two distinct membrane regions^20^ can lead to two greatly differing consequences for HER2, namely (a) sustained internalization, or (b) unaffected residency in the membrane. The drug effect as seen on the level of the whole cell population reflects the overall degree of ruffling in the bulk cell population, including the percentage of flat, resting, or dormant cells without membrane ruffles. Intra- and inter-cellular heterogeneity is known to play a crucial role in cancer and its therapeutic combat. By increasing the precision of the underlying datasets, consideration of heterogeneity can thus substantially improve the interpretation of observed drug effects in cancer cells through mathematical models.

## Methods and Materials

### Materials

SKBR3 cells were obtained from ATCC. Biotin and fluorescein conjugated Anti-HER2 Affibody Molecule (HER2-AFF-B, termed: (ZHER2:477)2) was purchased from Affibody AB, Bromma, Sweden. Dulbecco’s Phosphate Buffered Saline (DPBS), Dulbecco’s Modified Eagle’s Medium GlutaMAX^*TM*^ with high glucose and pyruvate (DMEM), Fetal Bovine Serum, certified, heat inactivated (FBS), Minimum Essential Medium Non-Essential Amino Acids (NEAA) 100× solution, Phosphate Buffered Saline (PBS) 10× solution, CellStripper, Normal Goat Serum (GS), and Quantum Dot (QD) Qdot 655 Streptavidin Conjugate (Strep-QD) were from Fisher Scientific GmbH, Schwerte, Germany. D-Saccharose, Glycine, biotin free and molecular biology grade Albumin Fraktion V (BSA), and Sodium Cacodylate Trihydrate were from Carl Roth GmbH + Co. KG, Karlsruhe, Germany. Electron microscopy grade Formaldehyde (FA) 16% solution was from Science Services GmbH, Munich, Germany. HPLC grade deionized Water, 0.01% poly-L-lysine (PLL) solution (mol wt 70,000–150,000), Sodium Tetraborate, Boric Acid, and Fibronectin-like Engineered Protein Polymer-plus (FLEP), were from Sigma-Aldrich Chemie Gmbh, Munich, Germany. 35 mm-cell culture dishes with uncoated glass bottom were from Greiner bio-one, Frickenhausen, Germany (CELLviewTM four compartments), and from ibidi, Munich, Germany (*μ*-Dish, with imprinted 50 or 500 *μ*m relocation grid).

### 35 mm-cell culture glass bottom dishes preparation for cell settlement

All glass bottom dishes were prepared for SKBR3 cell settlement by hydrophilization with Ar/O_2_ plasma for 5 min and subsequent incubation with 0.01% PLL, 500 mL per compartment, in four compartment dish, 2 mL for grid imprinted dishes without compartments, for 5 min at room temperature (RT). Afterwards, the dishes were rinsed twice in HPLC grade water, incubated in FLEP (15 *μ*g/mL in water, 3.7 *μ*g/cm^2^) for 5 min, rinsed twice in PBS, and kept in FBS supplemented cell medium until the cell suspension was ready (within 10–15 min).

### Cell culture and cell seeding on cell culture dishes

SKBR3 cells (human breast cancer cell line overexpressing HER2) were cultured in flasks (25 cm^2^) with cell media (DMEM, supplemented with 1% NEAA and 10% FBS), in a 5% CO_2_ atmosphere, at 37 °C. The cells were passaged twice a week at 60–80% confluency. Experiments were performed with cells between passages 6 to 22. The cells were harvested by rinsing in DPBS, dissociation with CellStripper (15–20 min, at 37 °C), and quenching with 4.5 mL of full cell media. 300–400 *μ*L, respect. 1.0–1.5 mL, of the SKBR3 cell suspension were added to each dish compartment, respect. to a grid imprinted dish, aiming to achieve 60–80% cell confluency on the next day. The next day, the cell media were exchanged to leave the cells under serum starvation overnight.

### Trastuzumab incubation and labeling of HER2

Cells grown to 70–90% confluency at day 2 after seeding were subjected to drug exposure and HER2 labeling, performed as described earlier^9^. To examine the effect of 10 *μ*g/ml trastuzumab (diluted in cell culture medium without serum) on the density of HER2 in the plasma membrane, the cells were exposed to trastuzumab for different periods of time, lasting between 3 min and 24 h, with or without subsequent chase periods in cell media without drug, lasting 2 or 5 h. All incubation and chase periods were performed at 37 °C in the CO_2_ incubator, and the live cells were never cooled to RT for more than a few minutes, and never cooled below RT. For investigating a possible recycling of internalized HER2 back to the plasma membrane, HER2 labeling with HER2-AFF-B was done before or after selected time periods of drug incubation, with or without subsequent chase periods. Before incubation with Strep-QD (5 nM in PBS including 1% BSA (BSA-PBS)) the cells were fixed with 3% formaldehyde (FA) and processed with rinsing and quenching steps as described earlier^9^.

### Direct interference contrast (DIC) and fluorescence microscopy

At the end of the drug exposure and HER2 labeling experiment, the cells were kept in BSA-PBS, and imaged in an inverted light microscope (DMI6000B, Leica, Germany) using two different channels, a DIC analyzer filter cube, to yield information about membrane borders and topography, such as membrane ruffles, and a filter cube with a 340–380 nm excitation and >420 nm emission window for QD emission. In most experiments a 20× objective was used, except for experiments designed to examine the influence of membrane ruffles, where a 40× objective was used. The imaging settings within the experimental groups and between the experiments were kept identical.

### Experiments to investigate the involvement of ruffles on the drug effect

After 2 days, including overnight serum starvation, SKBR3 cells that were grown in glass bottom dishes with imprinted grids, were imaged in CO_2_ independent medium for at least 15 min, at 37 °C, with time lapse light microscopy using the DIC channel, recording tiled images from randomly chosen areas of areas of 1 *μ*m^2^. The cells were then exposed for 1 h to trastuzumab with subsequent HER2-labeling as described above, followed by recording a second DIC and a QD emission image of the same areas. Control cells were processed similar, except that the drug was lacking during the 1 h drug incubation step. For the characterization of cellular subpopulations into ruffled/bulk or flat/resting cells, we determined the criterion for the later to be the complete absence or the presence of only a single ruffle per cell. All cells with more than one ruffle were sorted into the bulk cancer cell group. In addition, all cells sorted into the flat/resting cells group were checked in the time lapse data set to truly exhibit a resting behavior, i.e. lacking the appearance of more than 1 ruffle during the 15 min time window of time lapse imaging.

### Quantification of membrane-bound HER2 in control and in drug exposed cells

The recorded images were analyzed using the Leica Application Suite X (LAS-X) software, including a first visual screening of the images to exclude cells, which did not comply with our general criteria for analysis, i.e. cells of spherical shape and of small size^9^, i.e. <600 *μ*m^2^. In addition, cells that were only partially captured within the image frame were also excluded from further analysis. In general, all other cells were analyzed, except in experiments designed to examine the effect of membrane ruffles, where, due to the high number of imaged cells, the analyzed bulk cells were randomly determined until a similar number of cells was reached, comparable to the cell numbers in the other experiments. All cells cleared for further analysis were manually marked around their cell border, whereby the enclosed area represented the cell adherence area to the supporting glass. In this defined region of interest (ROI) the signals deriving from all QD-labeled HER2 on the respective cell were determined. The following values from the LAS-X analysis were used from the analyzed cell ROIs: (a) mean signal intensity per pixel, in the following referred to as luminosity, and (b) total HER2 signal intensity of the cell, i.e. the sum of all fluorescence pixel values within the ROI, as determined from the QD fluorescence channel and given as ‘grey values’. In addition, also the cell adherence area size was measured. To correct these values for unspecific background signal, created by the reflections of QD emitted light in the dishes, 3 to 4 regions of interest without cells were marked in every image. The average value of the mean signal intensity per pixel of these background ROIs was calculated for every image, and subsequently subtracted from the values of the cell ROIs in that image. In later experiments the individual background level was measured close to every single analyzed cell ROI and subtracted from the respective cell ROI values.

### Data preprocessing

The main dataset contained measurements for twelve conditions, differing in the length of treatment with trastuzumab and the time-point of labeling the receptors with the Affibody (before or after the drug treatment). About 200 cells were marked per condition providing information on their size (area) and luminosity. For each condition several pictures were acquired from different areas in the cell culture dish. All completely captured cells in the images were analyzed. Due to luminosity variations between the images also background areas were marked and measured for each image. Aim of the data pre-processing was to extract one comparable signal value, corrected for the background, for each condition. A detailed description of the signal pre-processing is given in the Supplementary Information. In summary, the signal per condition *s*_*c*_ was determined by a least squares estimation based on ${s}_{c,p}^{obs}={s}_{c}+{b}_{p}$ where the mean luminosity per cell was used as input for the observed signal ${s}_{c,p}^{obs}$ and the mean background luminosity per picture *b*_*p*_ corresponding to the observed background per picture ${b}_{p}^{obs}={b}_{p}$ was added.

### Mathematical Modeling

The receptor dynamics model was described by a set of reactions that were mathematically formulated as ordinary differential equations (ODEs), following mass-action kinetics. A model prediction was generated by solving the ODEs numerically for a given set of parameters, which consisted of initial amounts of the receptor states and the rate constants associated with the reactions. In general, the system was assumed to be in equilibrium at *t* = 0. This steady-state assumption induced relations between the rate constants and initial amounts that allowed to reduce the number of free parameters. The treatment of the cells with the Affibody or trastuzumab was implemented by switches that regulated the availability of these compounds. When available, the compounds could bind to the receptors leading to observability in case of the Affibody or altered reaction rates in case of trastuzumab.

The measured signal was proportional to the sum of Affibody bound receptors on the membrane. The proportionality factor was included in an observation function in order to allow the comparison of the simulated receptor time-courses with the measured time-resolved signal. For the optimization process, the model prediction *x*_*c*_ was scaled to match the observed signal *s*_*c*_, *s*_*c*_ = *k*_*s*_ · *x*_*c*_ + *ε*, where the scaling factor *k*_*s*_ was determined along with the other parameters of the model. In addition, we assumed that the measurement noise *ε* was normally distributed. For the presentation of the results, the data and model prediction were scaled inversely such that the predicted control condition corresponded to one at time point zero.

The free parameters were determined from the experimental data by the maximum-likelihood approach. The cost function is given by:

1 $$-2\,\mathrm{log}\,L(\theta )=\sum _{c}\,{(\frac{y({t}_{c},\theta )-{y}_{c}^{D}}{{\sigma }_{c}})}^{2}$$

where *y*(*t*_*c*_, *θ*) is the predicted observation at time point *t*_*c*_ for condition *c* given the parameters *θ*. The symbol ${y}_{c}^{D}$ denotes the data point for condition *c* at time point *t*_*c*_ and *σ*_*c*_ its uncertainty. Parameter estimates were obtained by minimizing eqn. (1) with respect to *θ*. The profile-likelihood method^74^ was applied to compute confidence intervals of the parameters and to test whether all parameters were identifiable given the experimental data, The model was reduced until all parameters are identifiable, following methods described elsewhere^75^.

All model analyses, parameter estimation and identifiable analysis were carried out using the dMod^76^ package for R. The package is available on the Comprehensive R Archive Network (CRAN). The structural non-identifiability of parameters due to scaling- and translation-symmetries was tested by a Lie-group approach^77^.

## Supplementary information

Supplementary Information
