# Supplementary material for: Mathematical modeling of drug-induced receptor internalization in the HER2-positive SKBR3 breast cancer cell-line
Source: Sci Rep. 2019 Sep 3;9:12709. doi: 10.1038/s41598-019-49019-x (PMC6722142; doi:10.1038/s41598-019-49019-x)
Supplement: Supplementary file 1 — Supplementary Information [file 41598_2019_49019_MOESM1_ESM.pdf]

Supplementary Information

Mathematical modeling of drug-induced receptor internalization in the HER2-positive SKBR3 breast cancer cell-line

Mirjam Fehling-Kaschek, Diana B. Peckys, Daniel Kaschek, Jens Timmer & Niels de Jonge

1 Visualization of trastuzumab-induced endocytosis of HER2

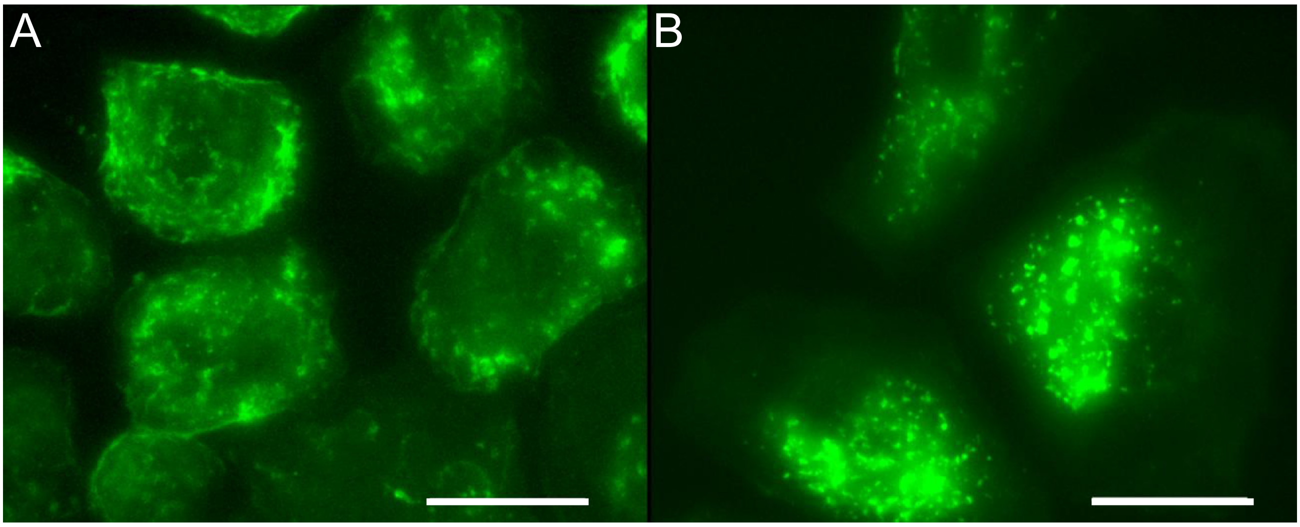

Figure S1. HER2 labeling of SKBR3 cells with FITC-conjugated Affibody. (A) The fluorescence image from a control group of SKBR3 cells shows the typical distribution of HER2 at the plasma membrane with higher densities in the ruffles. (B) SKBR3 cells treated for 60 min at 37°C with trastuzumab show signs of intracellular HER2 accumulation in vesicle-like structures, a remaining faint HER2 signal is also emitted from the plasma membrane. Shown are the maximum intensity projections of Z-stack images recorded with a 63× oil immersion objective. Scale bars: 20 μm.

To directly visualize the trastuzumab-induced endocytosis of HER2, a second control experiment was carried out in which the SKBR3 cells were first incubated with a fluorescein-conjugated Affibody against HER2, followed by incubation with trastuzumab for 1 h, as described in detail elsewhere[9]. The anti-HER2 Affibody conjugated with fluorescein was chosen to minimize the impact of label size and binding valency on the uptake process. This label is much smaller than the combined anti-HER2 biotin Affibody-QD label, and its monovalency excludes artificial clustering of labeled HER2, which would be possible when using multivalent Strept-QD in live cells. Cells in dishes were first incubated with 400 nM HER2-AFF-Fluo, for 30 min at 37°C, then exposed to the drug or to cell culture medium without FBS, for 1 h at 37°C, washed and fixed (as described above). Images were recorded with a 63× oil immersion objective from FA-fixed cells, using a FITC-filter cube. Fig. S1A shows a representative group of control cells with HER2 signals accumulating on ruffles and at the cell edges. In the drug treated cell group the majority of cells show HER2 signals in small vesicles (round bright shapes) and a remaining dimmer fluorescence signal on the plasma membrane (Fig. S1B).

A control experiment was carried out in order to exclude any HER2 down-regulation by the anti-HER2 Affibody. Two groups of control SKBR3 cells were subjected to the standard two-step labeling protocol using Affibody and

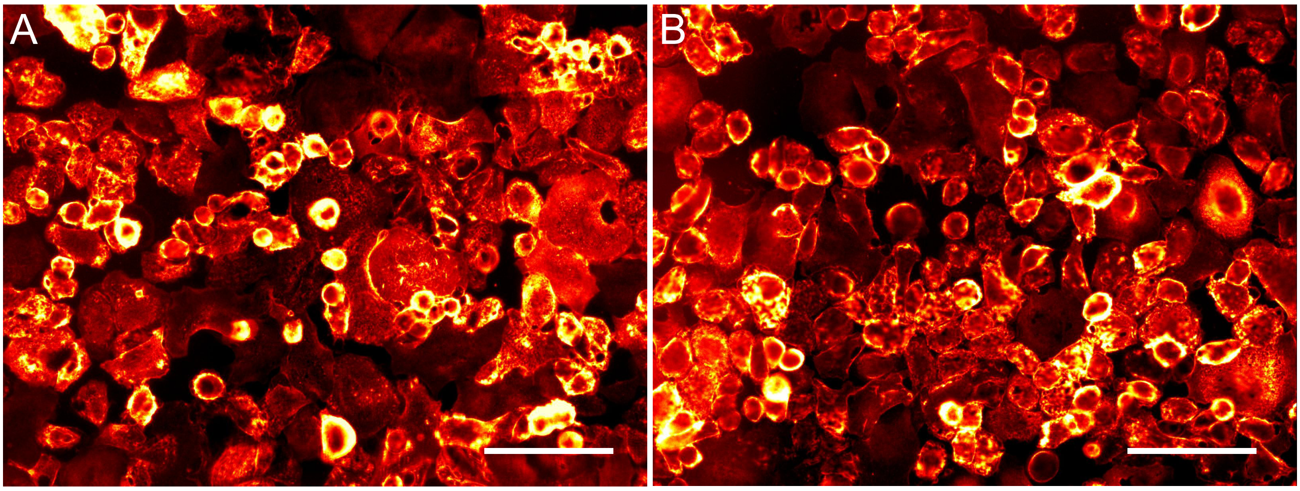

Figure S2. Control experiment showing that the anti-HER2 Affibody itself does not induce down-regulation of membrane-bound HER2. (A) HER2 fluorescence intensity of control cells after 10 min Affibody incubation of live cells, followed by fixation and QD-incubation, as performed for the control groups. (B) HER2 fluorescence signal of cells prepared similarly, but with an added chase lasting 60 minutes after the Affibody incubation and before fixation. The cells display the same average signal intensity as those from the control group, ruling out a significant HER2 internalization induced by the Affibody during the chase time. Both images were recorded and represented with the same settings. Scale bars: 100 μm.

quantum dots (QDs), whereby the second group had an extra chase lasting 60 minutes after the Affibody incubation and before fixation. The HER2 signal intensity did not significantly differ between the two groups, thus confirming that the Affibody did not led to HER2 internalization during the chase time, see Fig. S2.

A control experiment was performed to test the influence of low-temperature exposure on drug-induced HER2 internalization. For this purpose, SKBR3 cells were divided into 4 groups. The first 2 groups were processed as was done for our main data set, using a control group without drug incubation processed with the standard protocol HER2 labeling, and a corresponding cell group subjected to a 60 min drug exposure, after prior Affibody incubation, all steps performed at 37°C. The other two groups were processed with similar labeling and drug incubation protocols, but before, the control group was subjected to a 60 min cooling period at approximately 4°C by placing the sample containing dish on crushed ice, followed by a 60 min chase period at 37°C. The low-temperature drug group was treated similar, but incubated with trastuzumab during the cooling period. As can be seen in Fig. S3, already the 60 min cooling period on ice provoked dramatic changes in the membrane distribution of HER2, still visible after a subsequent 60 min chase at 37°C. Furthermore, when the drug was given to the cooled cells, the effect of HER2 internalization was severely attenuated compared to cells continuously kept at 37°C during drug incubation.

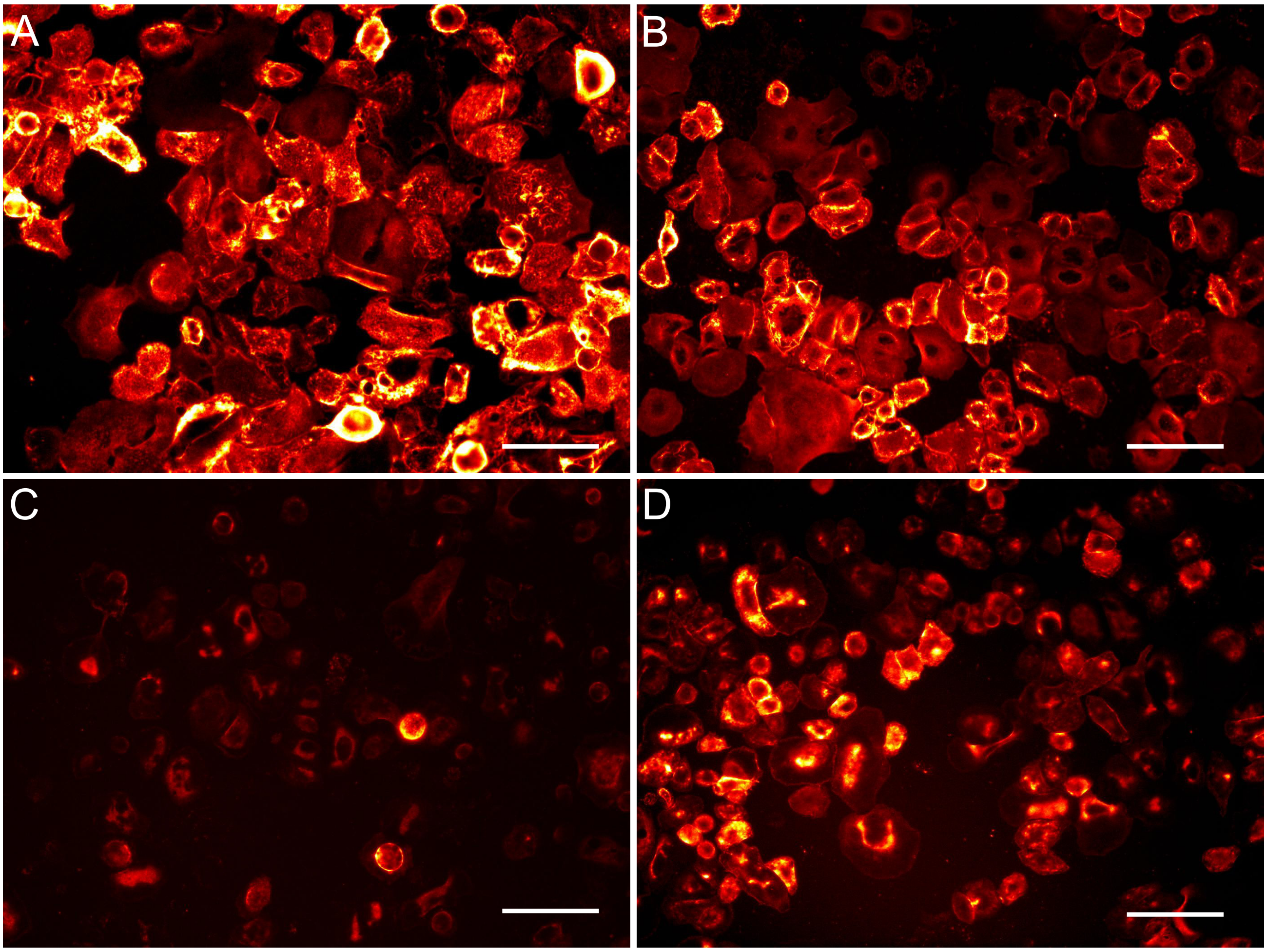

Figure S3. Effect of cooling on HER2 membrane distribution and trastuzumab induced internalization in SKBR3 cells. (A) Control cells after HER2 labeling via the standard protocol, that is live cell incubation with Affibody for 10 min at 37 °C, prior to fixation and QD labeling (no cooling). (B) Cells were kept for 60 min on ice in CO_2 independent medium, then chased at 37 °C for 60 min, followed by the standard HER2 labeling protocol. Many cells exhibit changes in HER2 distribution, mainly a fading of HER2 from central areas. (C) Cells were first labeled with Affibody (as in the standard protocol), then exposed to trastuzumab in CO_2 independent medium for 60 min, at 37 °C, followed by fixation and QD labeling. A marked reduction of membrane-bound HER2 is visible. (D) Cells were treated as those shown in C, but the 60 min incubation with trastuzumab was done at a temperature of approximately 4°C. These cells exhibit a smaller reduction of membrane-bound HER2 than the non-cooled cells shown in C. All images were recorded and represented with the same settings. Scale bars: 100 μm.

2 Data preprocessing

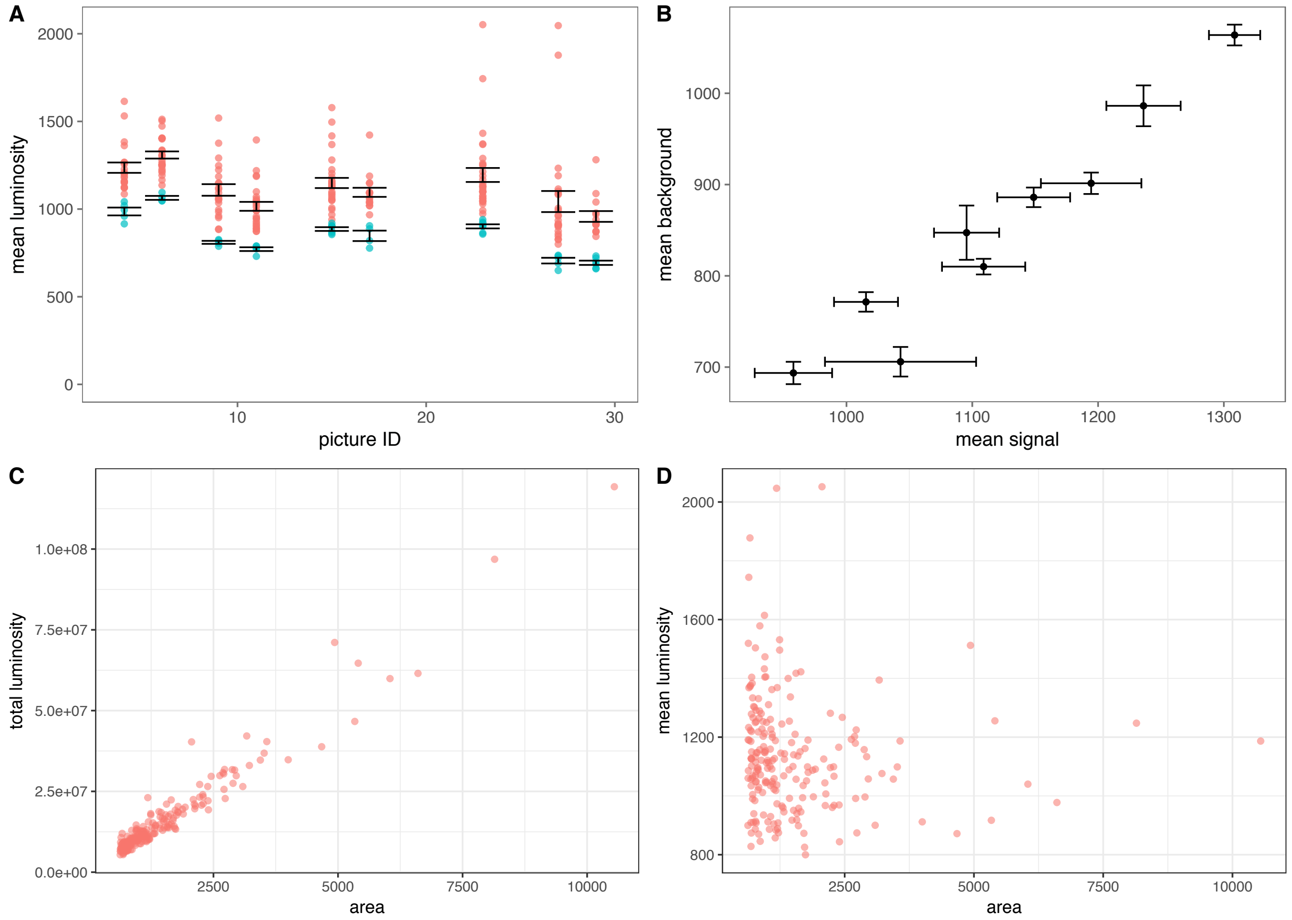

Figure S4. Data distributions for the condition with 20 min trastuzumab treatment. A) Mean cell luminosity (red) and background luminosity (blue) per picture with corresponding mean values (black), B) corresponding mean values per picture for the mean cell versus mean background luminosities, C) total cell luminosity versus cell size and D) mean cell luminosity versus cell size.

The experimental dataset contained images/pictures acquired for several different conditions, whereby each condition corresponds to a specific drug and Affibody treatment. The conditions are listed in Table 1. For each condition, several pictures were recorded and each image contained 20-30 cells. About five background measurements were made for each image. In Fig. S4A, the mean luminosities for the cells and background measurements per picture are shown exemplary for the condition with 20 min trastuzumab treatment. First, it is clearly visible that the background luminosity varies between the pictures. Second, a correlation of the signal with the background luminosities is apparent. This is further illustrated in Fig. S4B where the mean background measurements are plotted versus the mean signal luminosities for the same condition of 20 min trastuzumab treatment. Several sources can contribute to the observed background. The question is whether it is overlapping with the signal contribution in the measured cells, for example, due to backscattering of light from the walls of the dishes, or if it stems additionally from QDs adhering unspecifically on the coated, free cell surface areas of the dish. In the first case, the observed signal would be a sum of the true underlaying signal s and the background contribution, $s_{obs} = s + c \cdot b$ where the constant c can account for a damped or increased background contribution within the cells. In the second case, the background luminosity would reflect the general luminosity fluctuations between the pictures and would allow for a normalization of the signal via $s_{obs} = s \cdot c \cdot b$. Both cases may have contributed to

the observed signal background correlation shown in Fig. S4B. A distinction is possible via an inspection of the measured signal variances. As the variance of the background luminosities turned out to be small compared to the variance of the signal luminosities, the background variance was neglected for the following reason. In case of an additive signal-background model, the observed signal variance is independent of the background, while it correlates with the background for the multiplicative background. In Fig. S5, the signal variance is plotted versus the mean background contribution for all pictures of the dataset and the estimated correlation are shown. Following the above argumentation, the additive signal-background model was chosen.

Another decision was made concerning the signal luminosity. Either the total or the mean luminosity per cell can be taken as input for the mathematical modeling. The total luminosity is strongly correlated to the cell size (Fig. S4) and therefore an unwise choice if the areas of the selected cells fluctuate between conditions. On the other hand it would not be affected by a systematic change in cell size that could possibly be induced by trastuzumab. In contrast, the mean luminosity per cell would be affected by a systematic change in cell size but compensate for random fluctuations of the cell sizes between conditions. The mean cell areas for the different conditions of the main dataset are shown in Fig. S6. Random size fluctuations are visible that are not correlated to the trastuzumab treatment. In conclusion, the mean luminosity per cell was chosen as input for the modeling.

As a final step, the single cell measurements were condensed into one signal observation per condition. This was done via a maximum likelihood fit based on all single cell and background measurements. The signal per condition s_c was estimated via the underlying model $s_{c,p}^{obs} = log(s_c + c \cdot b_p)$ and $b_p^{obs} = log(b_p)$ from the log-transformed background measurements per picture b_p^{obs} and the log-transformed cell measurements per condition and picture $s_{c,p}^{obs}$ for a global scaling constant c. A constant global uncertainty was assumed for each the background and signal observation, corresponding to a relative uncertainty on non-logarithmic scale. The estimated scaling constant c was found larger than one $c = 1.15 \pm 0.1$, corresponding to a scenario where the background contribution was larger in the cells than in the areas surrounding the cells. In addition, the smallest signal value was found non-identifiable. Both issues were solved by fixing the constant to one, implying the assumption that the background contribution overlaying the cell signal was as large as the background contribution in the cell free areas.

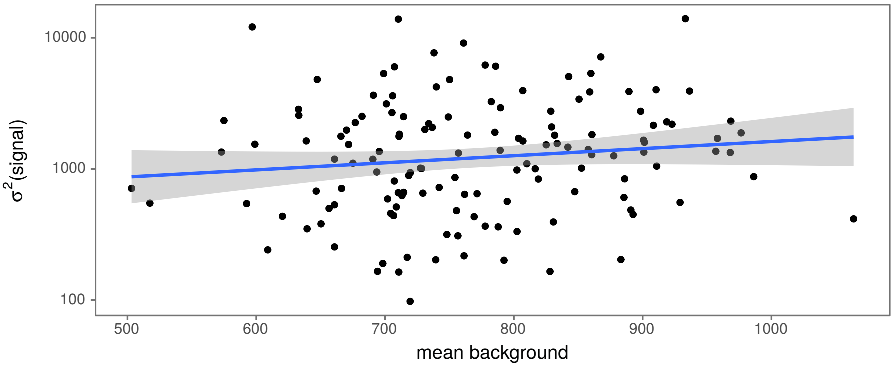

Figure S5. Variance of the mean luminosity of the cells as function of the corresponding background measurement. All measured conditions are included and each point corresponds to one picture.

3 Recycling Model

The model description and main results for the first model are given in section 2. The ordered values of the cost function $(-2\log(L))$ of the 300 best fits obtained from about 400 fits started with randomly distributed initial parameter values are shown in Fig. S7A. After constraining the model with literature values on the fraction of internal receptors and Affibody binding rate and neglecting the receptor synthesis, three solutions were found (see Fig. S7B) describing the data equally well. Parameter profiles for the three solutions are shown in Fig. S8. The main difference between the solutions was found for the k_{rec} parameter, which is found negligible for the second solution

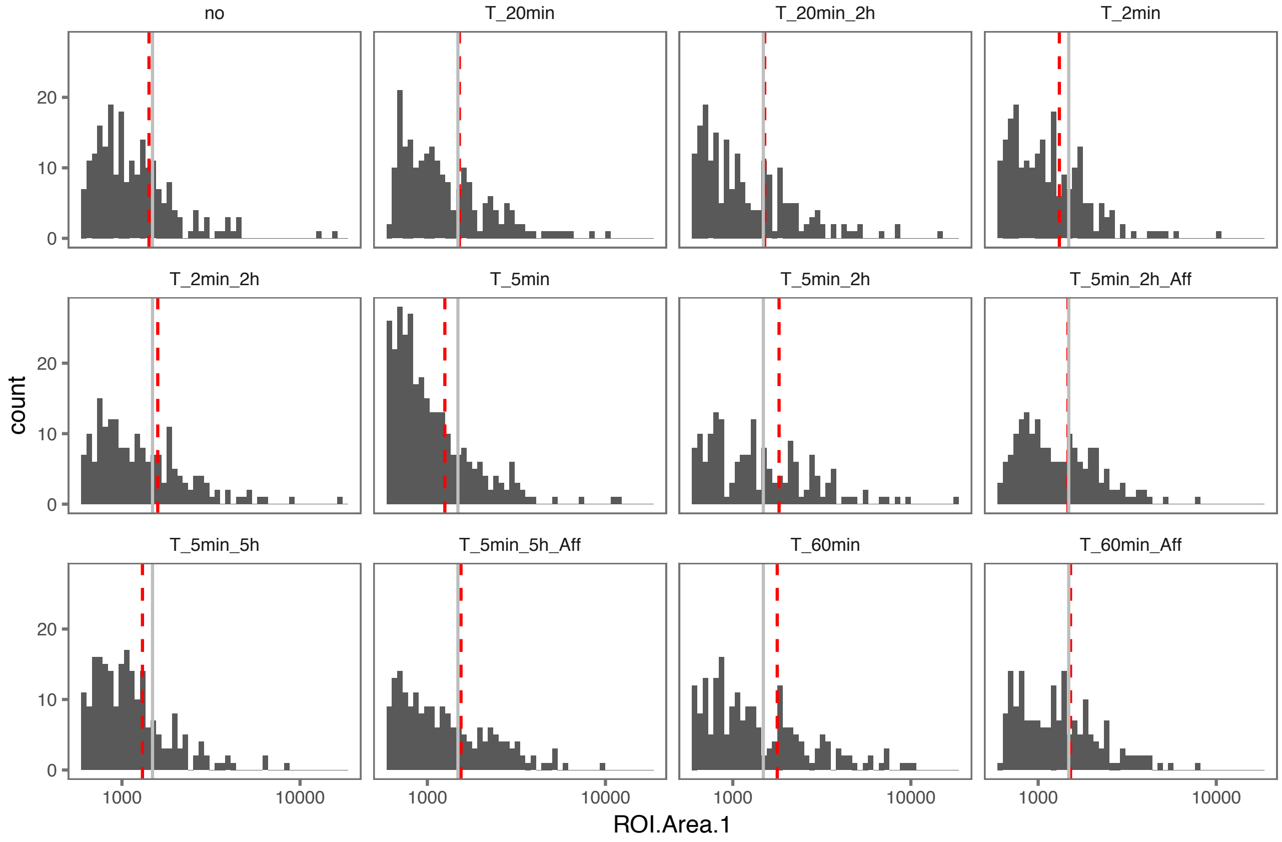

Figure S6. Cell size distributions for all conditions. The mean area per condition is shown as red dashed line and the mean area of all cells is shown as solid grey line.

but non-negligible for solutions 1 and 3. Solution 2 was chosen since it resulted in a smaller model after reducing negligible processes: as a final step the initial fraction of internalised receptors $N_i(t = 0)$ and the corresponding internalisation k_{int} and recycling k_{rec} processes were removed. The results are shown in Fig. 3. In Fig. S9, the predicted trajectories and fluxes are shown for solution 1 and 3, not applying the last step of model reduction. The result is comparable to the result of the reduced version of solution 2, particularly also large fluxes for the trastuzumab induced internalisation and recycling processes are found. Fig. S10 shows the parameter dependencies obtained from the parameter profile likelihoods of the final fit for the recycling model projected to the pairwise dependencies.

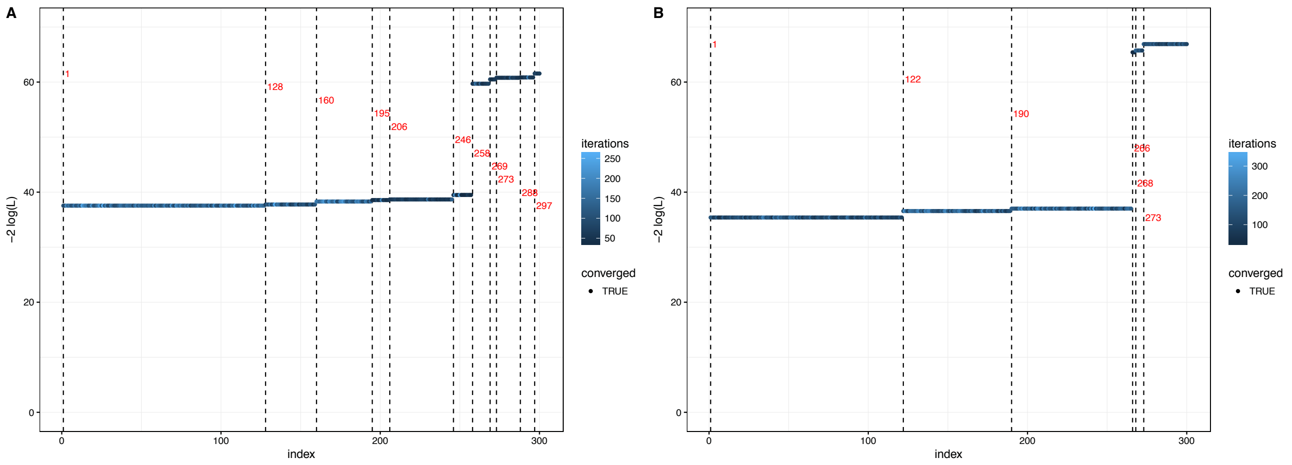

Figure S7. Ordered values of the 300 best converged fits before applying any model reductions A) and after applying the reductions up to removing the receptor production process B). Vertical lines separate the local optima with a 0.02 tolerance. Broad L2 parameter priors ($\sigma = 10$) were added to the cost function for each parameter to avoid technical difficulties for the optimization.

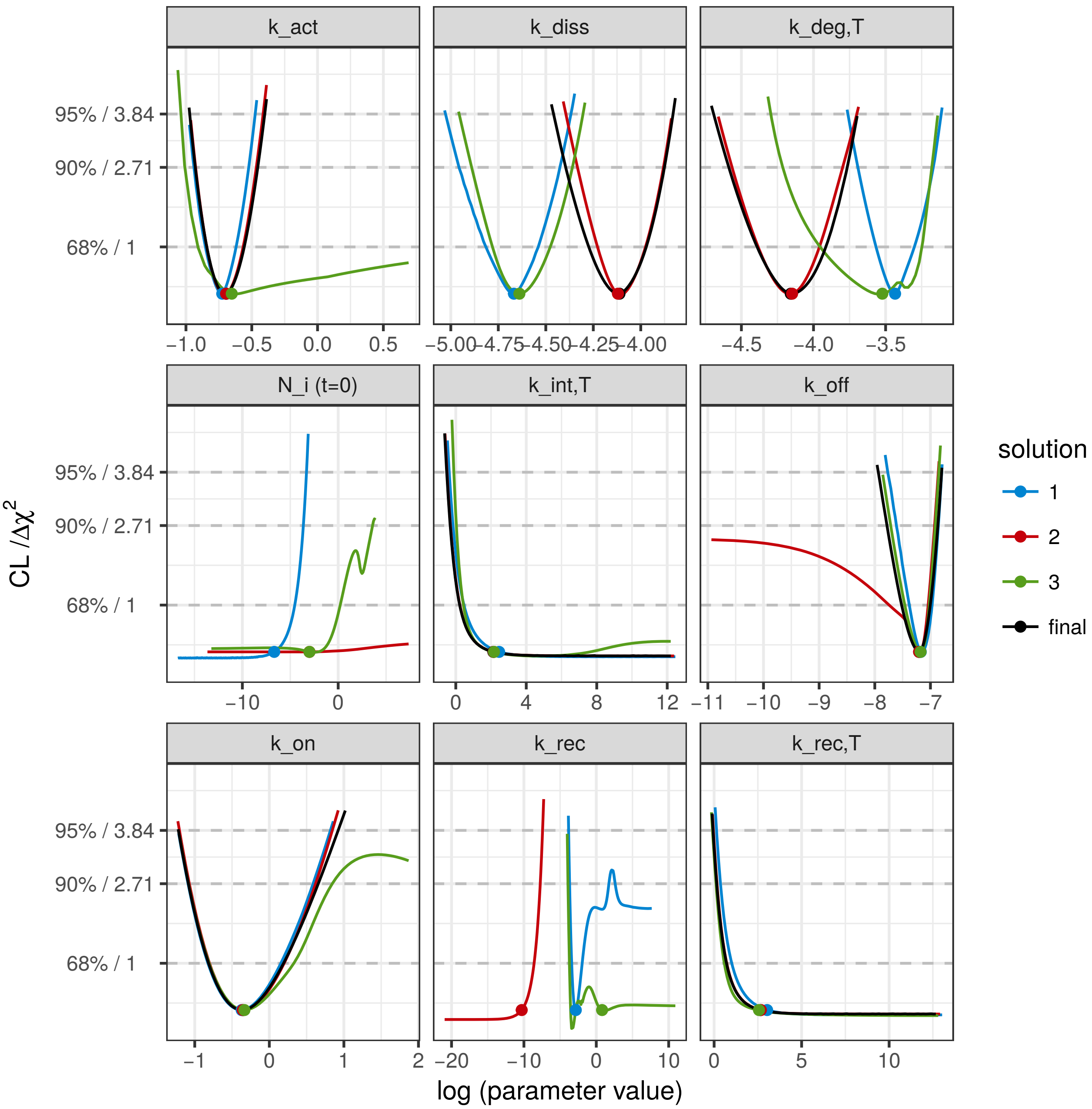

Figure S8. Parameter profiles for the recycling model. Only the data contribution to the objective function is included, not showing the parameter prior contributions. Solution 1-3 correspond to the three solutions found prior to the last model reduction and the final solutions corresponds to the model including also the reduction of neglecting k_{int} and k_{rec} for the non-activated receptors.

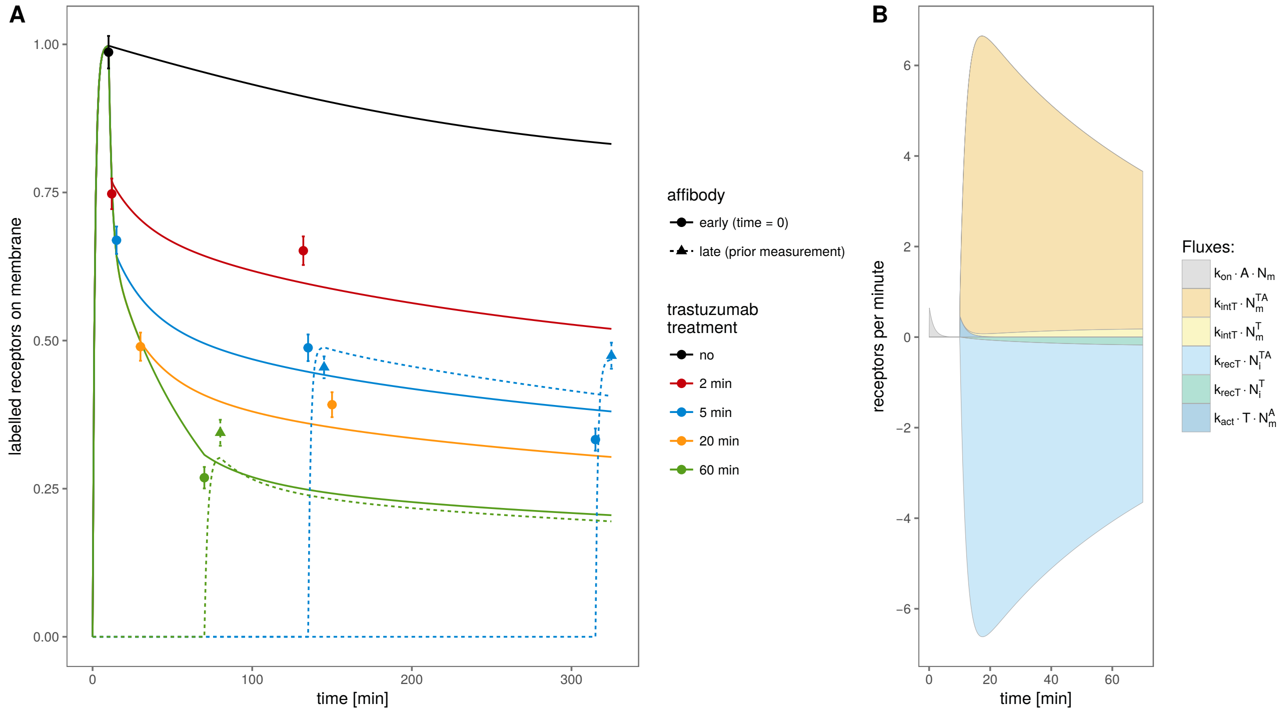

Figure S9. A A: Model trajectories with data points for solutions 1 and 3 of the recycling model, normalised by dividing by the scaling constant. B: Fluxes for the solutions 1 and 3 of the recycling model for the condition with 60 min trastuzumab treatment. The sign of the flux was chosen such that the backward processes (disassociation and recycling) are negative while all other processes give positive fluxes. Contributions of fluxes not shown were found below the visibility threshold.

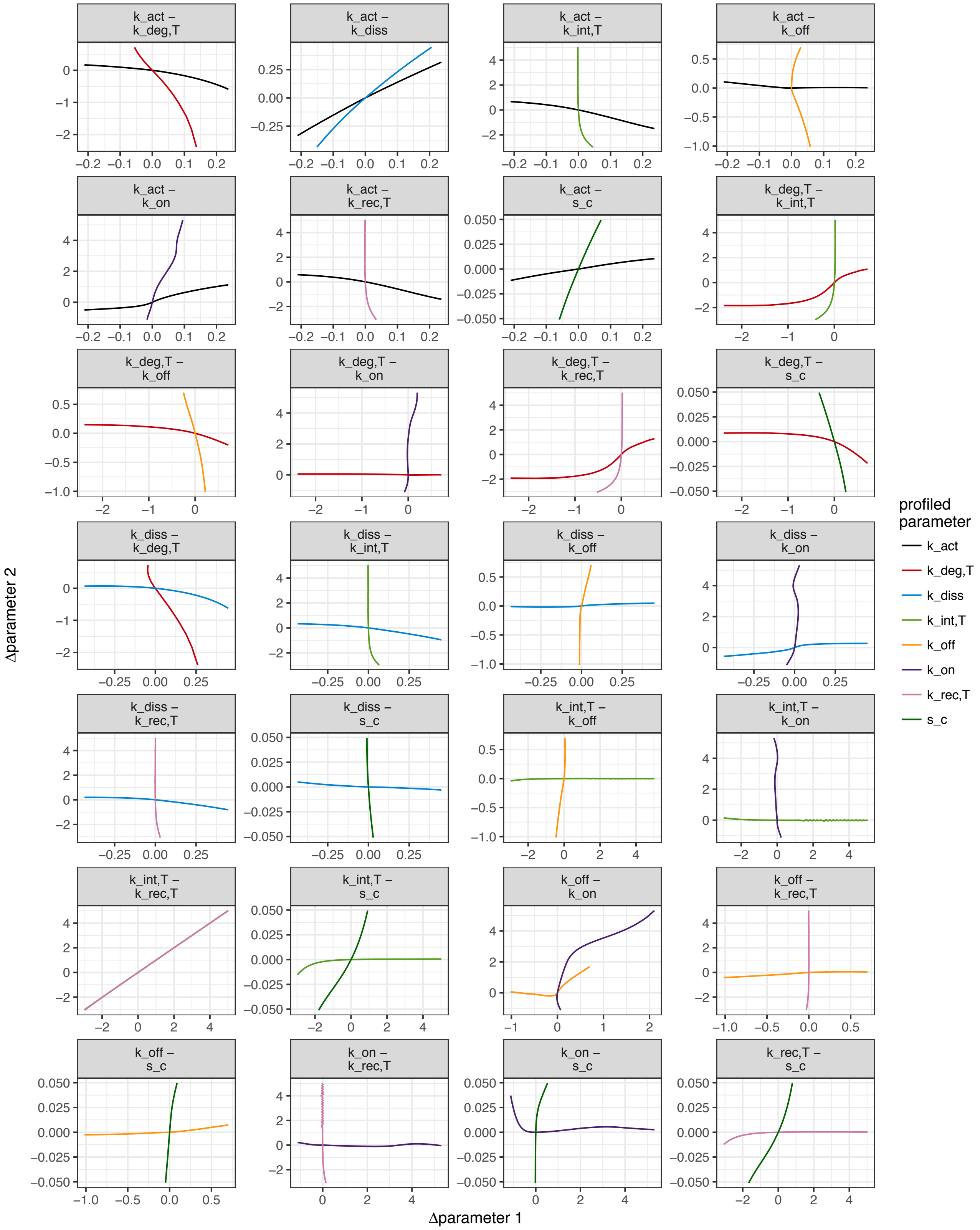

Figure S10. Pairwise parameter dependencies obtained from the parameter profiles of the final fit for the recycling model.
